# Targeted Inhibition of Colorectal Carcinoma Using a Designed CEA-Binding Protein to Deliver p53 Protein and TCF/LEF Transcription Factor Decoy DNA

**DOI:** 10.3390/ijms26209846

**Published:** 2025-10-10

**Authors:** Wen Wang, Xuan Sun, Geng Wu

**Affiliations:** State Key Laboratory of Microbial Metabolism, School of Life Sciences & Biotechnology, Shanghai Jiao Tong University, Shanghai 200240, China; vivi_w@sjtu.edu.cn (W.W.); sunshine.1997@sjtu.edu.cn (X.S.)

**Keywords:** colorectal carcinoma (CRC), p53, Wnt signaling pathway, TCF/LEF, transcription factor decoy (TFD), targeted delivery, de novo protein design, CEA, precision anticancer therapeutics, Max, p14ARF

## Abstract

Colorectal carcinoma (CRC) is characterized by mutations in p53 and the Wnt signaling pathway, and immunotherapy has shown limited efficacy in microsatellite-stable CRC. Here, CEABP1, a binding protein for the CRC biomarker carcinoembryonic antigen (CEA), was designed de novo through the AI-based computational generation methods RFDiffusion/ProteinMPNN and stringent in silico selection, for targeted delivery of purified p53 protein and transcription factor T-cell factor (TCF)/lymphoid enhancer-binding factor (LEF) transcription factor decoy (TFD) DNA into CRC cells. The cell-penetrating peptide (CPP) p28 was employed to deliver the p28-p53-CEABP1 protein, which significantly enhanced p53’s inhibition of CRC cell proliferation and xenograft tumor growth. Codelivery of the p14ARF protein together with p53 prolonged the effective antitumor duration of p53. In addition, the DNA binding domain of Max was fused with CPP and CEABP1 to deliver TCF/LEF TFD DNA, comprising concatenated consensus binding motifs for TCF/LEF and Max, into CRC cells to inhibit Wnt target gene transcription, leading to marked suppression of CRC cell proliferation and xenograft tumor growth. These findings paved the way for the development of precision anticancer therapeutics using designed binding proteins of tumor biomarkers for targeted delivery of tumor suppressor proteins and TFD DNA.

## 1. Introduction

Colorectal carcinoma (CRC) is the third most diagnosed cancer worldwide, with almost two million new cases and more than 903 thousand deaths each year [1,2]. Almost 50% of people may develop at least one benign intestinal tumor in their life [3]. CRC is commonly categorized into nonhypermutated and hypermutated CRC. Nonhypermutated CRC is usually microsatellite stable (MSS) but chromosome instable (CIN), accounting for 84% of CRC cases. In contrast, hypermutated CRC, which accounts for 16% of CRC cases, is generally characterized by microsatellite instability (MSI), with mutations in DNA mismatch repair (dMMR)-related proteins [4,5,6]. For hypermutated CRC with dMMR or high MSI, anti-PD-1/anti-PD-L1 immunotherapy has been proven to be effective. However, for the MSS type of CRC, which constitutes the majority of CRC cases, the efficacy of immunocheckpoint inhibitors is limited [7].

The occurrence of CRC is generally the result of multiple genetic mutations. First, mutations in the tumor suppressor p53 frequently occur in CRC, which usually happens in late adenoma or carcinoma stages [8,9]. Encoded by the *TP53* gene, the p53 protein is known as the “guardian of the genome” [10], and 50% of all cancer patients are found to have p53 mutations [11,12,13]. In 60% of nonhypermutated CRC cases, mutations in p53 were detected [4]. p53 functions mainly as a transcription factor, promoting the expression of p21^Cip1^ protein (encoded by the *cdkn1a* gene) to inhibit the cyclin–CDK complex and cause cell cycle arrest [14,15], as well as stimulating the expression of *bax*, *noxa*, and *puma* to lead to cancer cell apoptosis [16,17,18,19]. p53 also possesses functions independently of transcription [13]. The intracellular protein level of p53 is maintained at a low level by several E3 ubiquitin ligases, including Mdm2, ARF-BP1/Mule, COP1, and Pirh2, which polyubiquitinate p53 for degradation by the 26S proteasome [20,21,22,23,24]. p14ARF, which is another tumor suppressor, interacts with both Mdm2 and ARF-BP1/Mule to counteract their polyubiquitination of p53 so that p53 is prevented from degrading [25,26,27,28,29]. Second, more than 94% of CRC cases are correlated with mutations in proteins of the Wnt signaling pathway, such as biallelic inactivating mutations of *apc* [4], which typically occur in benign adenoma stages [9,30]. These mutations result in abnormally elevated levels of β-catenin in the cytoplasm, which translocates into the nucleus and interacts with the transcription factor T-cell factor (TCF)/lymphoid enhancer-binding factor (LEF), promoting the transcription of Wnt target genes such as *cyclin D1* and *c-myc* and leading to abnormal cell proliferation [31,32,33]. Other genes including *kras*, *pik3ca*, and *smad4* are also frequently mutated in CRC [4,6].

In current cancer therapy, only for mutations in oncoproteins do intervention measures exist, such as small molecule inhibitors, siRNAs, and PROTACs. On the other hand, for mutations in tumor suppressor proteins such as p53, there are no suitable therapeutics available. A promising strategy is to use cell-penetrating peptides (CPPs) [34,35,36,37,38] or other means [39,40,41] to deliver purified tumor suppressor proteins into tumor cells to inhibit cancer cell proliferation. However, the previous works suffered from a lack of specific targeting for cancer cells and the instability of the p53 protein because of the actions of multiple E3 ubiquitin ligases, including Mdm2 and ARF-BP1/Mule [11]. Therefore, anticancer therapeutics based on p53 protein delivery have not been widely developed.

In addition, although mutations in proteins often lead to excessive activation of the Wnt signaling pathway in colorectal cancer, there are currently no effective means to antagonize overactivated Wnt signaling [42]. The potential of nucleic acids as a therapeutic means to treat diseases is continuously being explored. The current nucleic acid-based therapeutics include antisense oligonucleotides (ASOs), aptamers, small interfering RNAs (siRNAs), CRISPR/Cas9-based gene editing, and transcription factor decoys [43,44,45]. In transcription factor decoy (TFD) technology, a short piece of double-stranded DNA, which represents the consensus binding site for the target transcription factor, is added to cells, where it competitively interacts with endogenous transcription factor proteins and prohibits the transcription of target genes [46,47,48]. However, due to the low efficiency and lack of specificity for delivering TFD DNA into cancer cells, this technology has not yet been widely applied in anticancer therapeutics.

In this work, the AI-guided protein design approach was employed to design an artificial protein CEABP1 de novo for specific recognition of the tumor biomarker of CRC, carcinoembryonic antigen-related cell adhesion molecule 5 (CEACAM5, abbreviated as CEA hereafter) [49,50]. This designed protein, CEABP1, was fused to p53 tagged with p28, a CPP derived from *Pseudomonas aeruginosa* [51,52,53,54], to specifically deliver purified p53 protein into CRC cells through endocytosis to stimulate the expression of p53 target genes, promote cell cycle arrest and apoptosis, and prevent CRC cell growth. The purified N-terminal domain of the p14ARF protein could also be delivered by CPP simultaneously with the p53 protein to prolong its lifetime and increase its effective duration. In addition, CEABP1 was fused with the DNA-binding domain of human Max protein fused with a CPP to specifically deliver TCF/LEF TFD DNA, in which a TCF/LEF-binding sequence was concatenated with the Max-binding sequence, into CRC cells to competitively occupy the endogenous TCF/LEF in CRC cells, repress overactivated Wnt signaling, and suppress CRC cell proliferation. These studies will contribute to further development of precision anticancer therapy based on using tumor biomarker binding proteins to deliver purified intracellular tumor suppressor proteins and TFD DNA.

## 2. Results

### 2.1. Delivery of Purified p28-p14ARF Protein Prolonged the Effective Duration of Delivered p53 Protein and Increased Its Antitumor Effect

**Figure 1:**
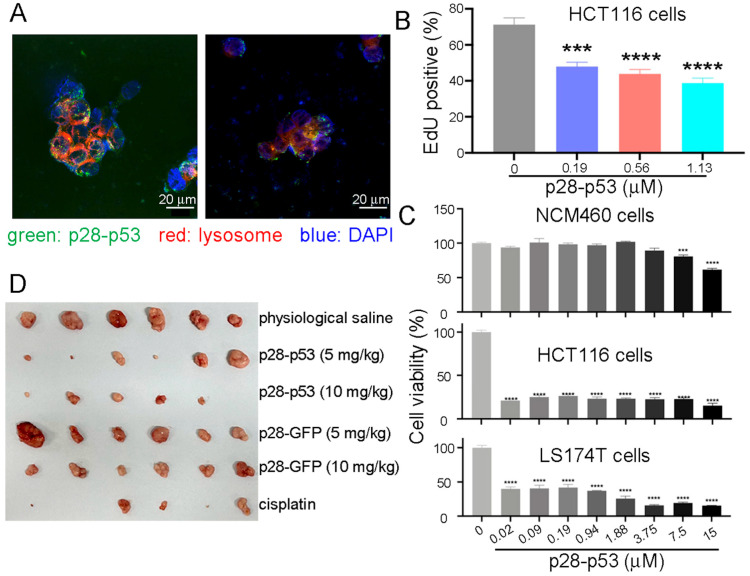
Delivery of purified p28-p53 protein inhibited CRC cell proliferation and xenograft tumor growth. (**A**) Confocal microscopy images showed that after delivery into HCT116 cells, the internalized p53 protein tagged with GFP and the cell-penetrating peptide p28 escaped from lysosomes and were localized in the nuclei. (**B**) Delivery of purified p28-p53 protein into HCT116 CRC cells inhibited cell proliferation, as revealed by the ethynyl deoxyuridine (EdU) incorporation assay. The error bars represent the standard error of the mean (SEM), which was determined via two-way ANOVA. (**C**) Delivery of the p28-p53 protein did not cause as much inhibition on the cell proliferation rate of NCM460 normal colonic epithelial cells as on that of HCT116 and LS174T CRC cells, as revealed by the CCK-8 cell proliferation assay. (**D**) Treatment of HCT116 cell subcutaneous xenograft tumor-bearing mice with purified p28-p53 protein decreased tumor growth. Representative images of HCT116 cell xenograft tumors at the endpoint of the experiment were shown; the experiment included the following: physiological saline (*n* = 6), 5 mg of p28-p53 protein per kg of mice (*n* = 6), 10 mg of p28-p53 protein per kg of mice (*n* = 5), 5 mg of p28-GFP protein per kg of mice (*n* = 6), 10 mg of p28-GFP protein per kg of mice (*n* = 6), and 5 mg cisplatin per kg of mice (*n* = 5). Administration started when the tumors grew to 70 mm^3^ in volume. Statistical significance is denoted as follows: *** *p* < 0.001, **** *p* < 0.0001.

p53 proteins fused with different CPPs, including pep1, penetratin (pene), p28, or TAT, were expressed and purified to homogeneity (Figure S1A) and then examined for their ability to suppress CRC cell proliferation and xenograft tumor growth. Among the different CPP-p53 proteins, p28-p53 exhibited the strongest suppression of CRC cell proliferation (Figure S1B). We confirmed that the purified GFP-tagged p28-p53 protein could be efficiently delivered into HCT116 CRC cells, with a fold change in the GFP intensity of approximately 90% (Figure S2). The delivered p28-p53 protein escaped from lysosomes and localized to the nuclei of HCT116 cells (Figure 1A) and was demonstrated to increase the expression of *cdkn1a* (Figure S3A) and *bax* (Figure S3B) in both HCT116 and LS174T CRC cells, as well as trigger the G0/G1 phase cell cycle arrest (Figure S4A) and apoptosis (Figure S4B). Delivery of the purified p28-p53 protein into HCT116 cells effectively inhibited cell proliferation in a dose-dependent manner, as revealed by the ethynyl deoxyuridine (EdU) incorporation assay (Figure 1B). Importantly, delivery of the p28-p53 protein did not cause much deceleration of the cell proliferation of the NCM460 normal colonic epithelial cells in most of the concentration range used. In contrast, delivery of the p28-p53 protein prominently caused inhibition of the cell proliferation of HCT116 and LS174T CRC cells at all of the concentrations used in the same CCK-8 cell proliferation assay (Figure 1C). In addition, in an in vivo study using immunocompromised athymic nude mice bearing HCT116 cells xenograft tumors, purified p28-p53 (Figure 1D and Figure S5), but not p28-GFP (Figure 1D and Figure S5) or p53 without CPP (Figure S6), exhibited potent inhibition of xenograft tumor growth.

**Figure 2:**
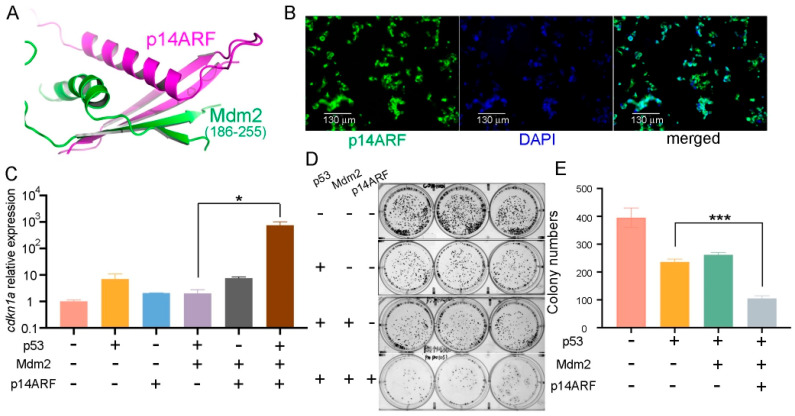
Delivery of purified p28-p14ARF protein strengthened the suppressive effects of the delivered p53 protein on CRC cell proliferation. (**A**) The N-terminal domain p14ARF (residues 1–63) folds into two β strands and an α helix and interacts with the middle part of human Mdm2 (residues 186–255), as predicted by Alphafold 3. (**B**) Purified GFP-tagged p28-p14ARF (residues 1–63, abbreviated as p28-p14^ARF^) protein was localized in the nuclei after delivery into HCT116 CRC cells. Scale bars: 130 μm. (**C**) Delivery of the p14ARF protein counteracted the inhibition of p53 by Mdm2 and drastically increased the expression of the p53 target gene *cdkn1a*. Purified p28-p53 and p28-p14ARF proteins were delivered while an Mdm2-encoding plasmid was transfected into HCT116 cells, and qRT-PCR analysis was subsequently performed. (**D**) Colony formation assays revealed that the purified p14ARF protein further increased the anti-proliferative effect of the purified p28-p53 protein on HCT116 cells. (**E**) Quantification of the colony formation assay results from (**D**). Statistical significance is denoted as follows: * *p* < 0.05, *** *p* < 0.001.

p14ARF prevents Mdm2 from downregulating p53 by regulating its nucleocytoplasmic shuttling and ubiquitination and also directly binds to and inhibits the E3 ubiquitin ligase ARF-BP1/Mule. Thus, the possibility of using delivered p14ARF protein to prolong the effective duration of delivered p53 protein in CRC cells was explored. Alphafold 3 was used to predict the human p14ARF-Mdm2 complex structure, which showed that the highly conserved N-terminal 63 residues of p14ARF (Figure S7) folded into two β-strands and an α-helix and interacted with a middle fragment of Mdm2 and residues 186–255 (Figure 2A, Figure S8). The complex between p14ARF (1–63) and Mdm2 (186–255) remained stable during the molecular dynamics simulation process (Movie S1). Therefore, the N-terminal residues 1–63 of p14ARF (abbreviated as p14ARF hereafter) were employed in the following study.

The purified p28-p14ARF protein was found to be localized in the nuclei after delivery into HCT116 cells (Figure 2B) and was found to indeed antagonize the inhibitory effect of Mdm2 on p53 and caused a dramatic increase in *cdkn1a* expression when simultaneously delivered together with p53 (Figure 2C). Furthermore, both the colony formation assay (Figure 2D,E) and the CCK-8 assay (Figure S9) results showed that delivery of purified p28-p14ARF protein enhanced the anti-proliferative effect of the delivered p53 protein in HCT116 cells, presumably as a result of antagonizing Mdm2 and ARF-BP1/Mule and resulting in stabilization of p53.

**Figure 3:**
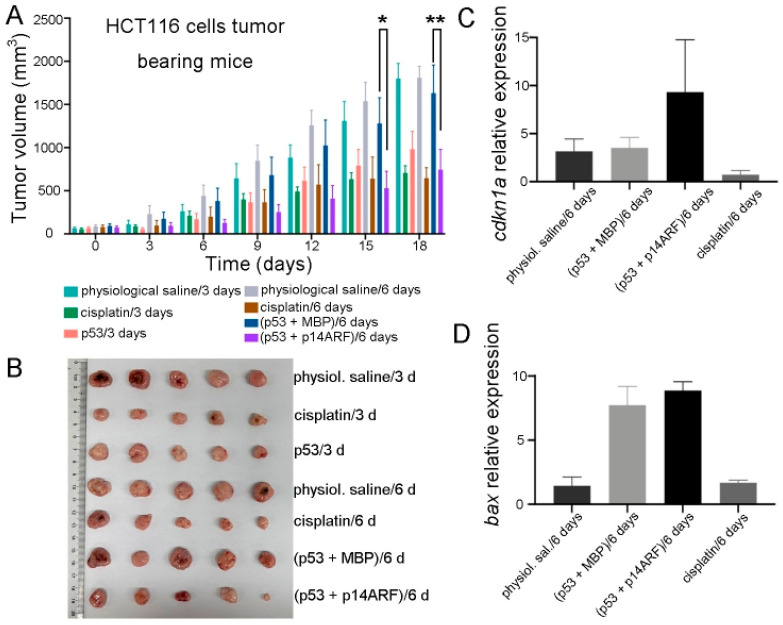
Delivery of purified p28-p14ARF protein prolonged the effective duration of the delivered p53 protein and further suppressed xenograft tumor growth in mice. (**A**) Compared with the administration of p28-p53 and MBP proteins once every 6 days which did not suppress tumor growth, the administration of p28-p53 and p28-p14ARF proteins once every 6 days substantially reduced the tumor growth rate in HCT116 cell subcutaneous xenograft mice. The tumor growth curves of the following groups receiving different treatments were shown: physiological saline every 3 days (*n* = 5), 5 mg cisplatin per kg of mice every 3 days (*n* = 5), 5 mg of p28-p53 protein per kg of mice every 3 days (*n* = 5), physiological saline every 6 days (*n* = 5), 5 mg cisplatin per kg of mice every 6 days (*n* = 5), 5 mg of p28-p53 and p28-MBP each per kg of mice every 6 days (*n* = 5), and 5 mg of p28-p53 and p28-p14ARF each per kg of mice every 6 days (*n* = 5). Administration started when the tumors grew to 100 mm^3^ in volume. Statistical significance is denoted as follows: * *p* < 0.05, ** *p* < 0.01 (**B**) Representative images of HCT116 cell xenograft tumors at the endpoint of the experiment. (**C**) qRT-PCR analysis revealed that treatment with purified p28-p53 and p28-p14ARF proteins every 6 days increased the mRNA expression of *cdkn1a* in the tumor, whereas treatment with purified p28-p53 and MBP proteins every 6 days had no effect. (**D**) qRT-PCR analysis revealed that treatment with purified p28-p53 and p28-p14ARF proteins every 6 days increased the mRNA expression of *bax* in tumors.

As a further analysis, immunocompromised athymic nude mice bearing HCT116 cell xenografts were injected every 6 days with purified p28-p53 and p28-p14ARF proteins or with purified p28-p53 and maltose-binding protein (MBP) as a control. Administration of the p28-p53 and MBP every 6 days did not slow down the progression of HCT116 xenograft tumors, in contrast with treatment with the p28-p53 protein every 3 days, which is likely due to the short half-life of the p53 protein in cells resulting from the negative regulation of E3 ubiquitin ligases. On the other hand, with codelivery of the p28-p14ARF protein, extending the dosing interval of p28-p53 to six days still resulted in considerable inhibition of subcutaneous HCT116 tumor growth in mice (Figure 3A,B and Figure S10A). Moreover, treatment with the p28-p14^ARF^ and p28-p53 proteins did not affect the body weights of the mice (Figure S10B). Importantly, codelivery of p28-p14ARF protein significantly enhanced p53-mediated upregulation of the downstream target genes *cdkn1a* (Figure 3C) and *bax* (Figure 3D) within the tumor tissue.

### 2.2. De Novo Design of Binding Proteins to Specifically Recognize the CRC Biomarker CEA

**Figure 4:**
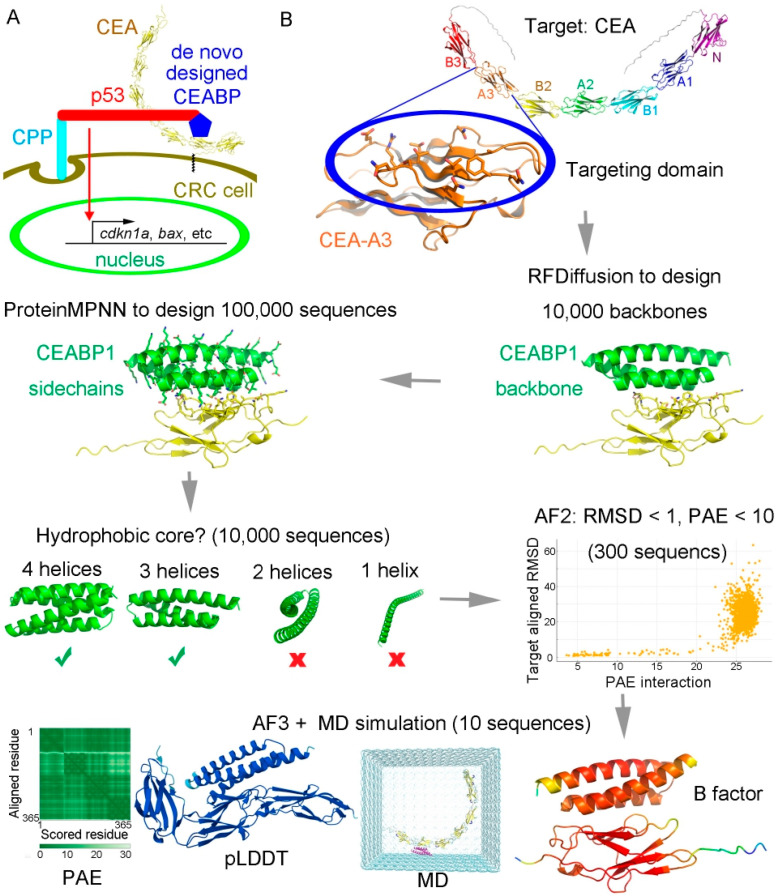
The strategy of using a de novo-designed CRC biomarker CEA binding protein to fuse with p28-p53 to facilitate specific targeted inhibition of CRC cells. (**A**) Scheme using a de novo-designed CEA binding protein CEABP to fuse with p53 tagged with the cell-penetrating peptide (CPP) p28, so as to specifically target CRC cells for inhibition. (**B**) The A3 or B2 domain of CEA was selected for specific targeting. The complete target CEA is shown at the top. The residues on one surface of CEA-A3 or CEA-B2 (highlighted in a blue oval) were chosen as “hotspots” for targeting binder design. RFDiffusion was employed to design the backbone scaffolds of binding proteins for CEA-A3 or CEA-B2, during which process 10,000 backbones were generated. ProteinMPNN was then employed to design the sequences, and 100,000 sequences were generated. Generated binders were filtered based on whether they contained hydrophobic cores. Designed proteins with four or three helices were selected, whereas those with two helices or only one helix were discarded. Alphafold2 was then employed to rapidly discard models that failed to recapitulate tight binding geometry. A molecular dynamics (MD) simulation for conformational sampling was performed, followed by Alphafold 3 rescoring to identify false positives that passed Alphafold 2 but were unstable during MD or had low predicted alignment error (PAE) or predicted local distance difference test (pLDDT) scores calculated by Alphafold3.

A goal of continuous effort in cancer drug discovery is to specifically target tumor cells without harming normal cells. To achieve this goal, de novo protein design technology was applied to design a binding protein for the extracellular domain of a membrane CRC biomarker. Carcinoembryonic antigen-related cell adhesion molecule 5 (CEACAM5, abbreviated as CEA hereafter) is a well-known tumor biomarker of colorectal cancer that is highly expressed on the membranes of CRC cells [49,50]. Alphafold 3 was employed to predict the structure of the full-length CEA, which showed that it consists of seven extracellular immunoglobulin (IgG) domains, which were called the N, A1, B1, A2, B2, A3, and B3 domains from distal to proximal to the cell membrane in the previous literature. As the N, A1, and B1 domains are relatively too far away from the cell membrane and the B3 domain is too close, the A3 and B2 domains of CEA were selected as the recognition targets of artificially designed proteins. Hence, our plan was to employ a designed CEA-binding protein (CEABP) to fuse with the p28-p53 protein in order to specifically recognize CRC cells in vivo to deliver the p53 protein in a targeted manner to prevent CRC tumor growth (Figure 4A).

**Figure 5:**
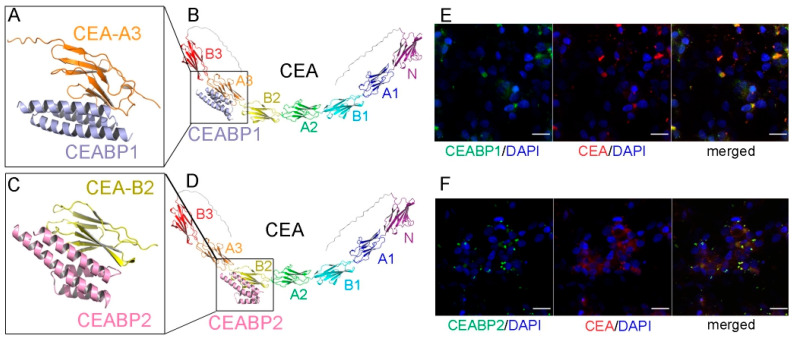
The de novo-designed CEABP1 and CEABP2, which specifically recognize the A3 and B2 domains of the CRC marker CEA, respectively. (**A**) The structure of CEABP1, the de novo-designed binding protein for CEA-A3, in complex with the extracellular A3 domain of CEA, as predicted by Alphafold 3. (**B**) The binding site of CEABP1 on the full-length CEA protein. (**C**) The structure of CEABP2, the de novo-designed binding protein for CEA-B2, in complex with the extracellular B2 domain of CEA, as predicted by Alphafold 3. (**D**) The binding site of CEABP2 on the full-length CEA protein. (**E**) CEABP1 colocalized with CEA on the cell membrane of the CEA-expressing CRC cell line LS174T. LS174T cells were incubated with purified GFP-tagged CEABP1 protein for 6 h before immunofluorescence staining. Scale bars, 20 μm. (**F**) CEABP2 colocalized with CEA on the cell membrane of LS174T cells.

RFDiffusion, which is based on diffusion models, was utilized to design the main-chain backbone scaffolds [55], and ProteinMPNN, which uses graph neural networks and a message-passing mechanism to integrate spatial and energy information [56], was employed to design the protein sequences. Using this scaffold-sequence two-step approach, binding proteins for the CEA-A3 domain (Figure S11) were designed and stringently selected in silico based on multiple criteria, including the presence of a stable hydrophobic core, the degree of agreement between the designed structure and Alphafold-predicted structure, and the stability during molecular dynamics simulation (Figure 5B).

One of them, named CEA-binding protein 1 (CEABP1; Figure 5A,B, Figures S12A and S13A,B), was predicted to possess high thermal stability, high binding affinity for CEA-A3, high solubility, and low immunogenicity, as verified by the epitope prediction tool in the Immune Epitope Database. Similarly, a binding protein for the CEA-B2 domain, CEABP2, was designed via RFDiffusion and ProteinMPNN (Figure 5C,D, Figures S12B and S13C,D). A molecular mechanics-generalized Born surface area (MM-GBSA) calculation of binding free energy was performed, and the ΔG value for CEABP1 binding to CEA was calculated to be −33.9252 ± 1.3432 kcal/mol, and that for CEABP2 binding to CEA was −29.4563 ± 1.0275 kcal/mol. The GFP-tagged CEABP1 and CEABP2 proteins were expressed in *E. coli*, purified, and were found via the immunofluorescence assay to colocalize with CEA on the cell membrane of LS174T CRC cells, which express high levels of CEA (Figure 5E,F).

### 2.3. Specifically Targeting CRC Cells with p28-p53-CEABP1 Resulted in Considerably Higher Suppression of CRC Cell Proliferation and Xenograft Tumor Growth

**Figure 6:**
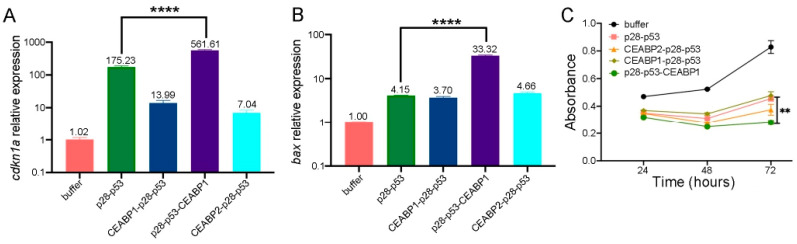
Specific targeting of CRC cells by fusing CEABP1 to the C-terminus of p28-p53 enhanced its ability to suppress CRC cell proliferation. (**A**) qRT-PCR analysis revealed that fusing CEABP1 to the C-terminus of p28-p53 was most effective at increasing the ability of the delivered p53 protein to promote *cdkn1a* transcription in LS174T cells. The relative *cdkn1a* expression levels are indicated above the columns. (**B**) The p28-p53-CEABP1 protein was most effective at increasing the transcription of *bax* when it was delivered into LS174T cells. The relative *bax* expression levels are indicated above the columns. (**C**) Compared with delivery of p28-p53, delivery of the purified p28-p53-CEABP1 protein more strongly inhibited the proliferation of LS174T cells, as shown by the CCK-8 assay. Statistical significance is denoted as follows: ** *p* < 0.01, **** *p* < 0.0001.

The designed CEABP1 or CEABP2 protein was fused either to the N- or C-terminus of the p28-p53 protein. With the exception of fusing CEABP2 to the C-terminus of p28-p53, which failed to yield detectable protein expression, the CEABP1-p28-p53, p28-p53-CEABP1, and CEABP2-p28-p53 proteins were purified to homogeneity and delivered to the CEA-expressing LS174T CRC cell line to examine the transcription of p53 target genes via the qRT-PCR assay. Among the three fusion proteins examined, delivery of the purified p28-p53-CEABP1 protein significantly enhanced the transcription of the p53 target genes *cdkn1a* (with a 3.2-fold increase, Figure 6A) and *bax* (with an 8.0-fold increase, Figure 6B) in LS174T cells compared with the delivery of p28-p53. In addition, delivery of the p28-p53-CEABP1 protein resulted in even stronger suppression of LS174T cell proliferation than delivery of the p28-p53 protein in the CCK-8 assay (Figure 6C).

**Figure 7:**
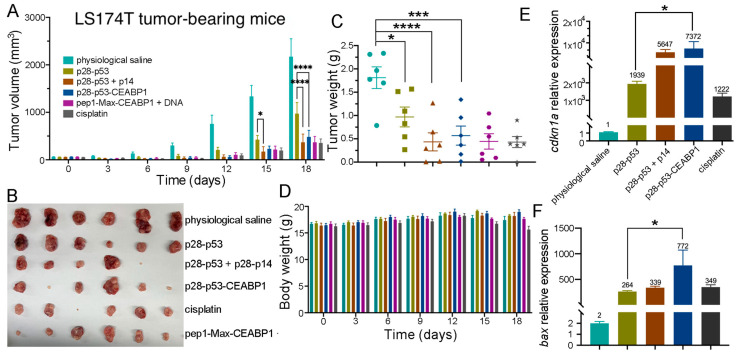
Specific targeting of CRC cells by fusing CEABP1 to the C-terminus of p28-p53 considerably increased its inhibition of xenograft tumor growth in mice. (**A**) Treatment with the purified p28-p53-CEABP1 protein inhibited LS174T cell subcutaneous xenograft tumor growth more effectively than p28-p53. The tumor growth curves of the following groups receiving different treatments are shown: physiological saline (*n* = 6), 5 mg of p28-p53 protein per kg of mice (*n* = 6), 5 mg of p28-p53 and p28-p14ARF each per kg of mice (*n* = 5), 5 mg of p28-p53-CEABP1 per kg of mice (*n* = 6), 8.28 mg of p28-p53-CEABP1 and TCF/LEF TFD DNA (molar ratio 3:1) per kg of mice (*n* = 6), and 5 mg cisplatin per kg of mice (*n* = 6). Administration started when the tumors grew to 100 mm^3^. (**B**) Representative images of LS174T cell xenograft tumors at the experimental endpoint (day 18). (**C**) LS174T tumor weights at the endpoint. The color scheme for the different groups is the same as that in (**A**). (**D**) Treatment with p28-p53-CEABP1 did not cause perceptible changes in mouse body weight during the experiment. The color scheme for the different groups is the same as that described in (**A**). (**E**) qRT-PCR analysis revealed that, compared with the absence of CEABP1, the injection of purified p28-p53-CEABP1 substantially increased the *cdkn1a* transcript level in the tumor tissue. (**F**) qRT-PCR analysis revealed that p28-p53-CEABP1 drastically increased *bax* mRNA expression compared with p28-p53 in tumors. The color scheme for the different groups is the same as that in (**E**). Statistical significance is denoted as follows: * *p* < 0.05, *** *p* < 0.001, **** *p* < 0.0001.

To further validate the tumor-suppressive effect of p28-p53-CEABP1 in vivo, we established a subcutaneous LS174T xenograft tumor model in nude mice. Consistent with the cell assay results, tail vein injection of the p28-p53-CEABP1 protein demonstrated substantially greater inhibitory efficacy (*p* < 0.0001) against LS174T tumor growth than did p28-p53 protein treatment (Figure 7A,B). At the end of the experiment, the tumor weight of the group of mice receiving p28-p53-CEABP1 protein was considerably lighter than that of the group receiving p28-p53 treatment (Figure 7C), whereas the body weights of the mice did not seem to show visible changes throughout the experiment (Figure 7D). In addition, hematoxylin and eosin staining analysis of harvested major organs (heart, lung, liver, kidney, and spleen) revealed no detectable histopathological abnormalities in the mice that received p28-p53, p28-p53 together with p14, or p28-p53-CEABP1 treatment (Figure S14). The inhibitory effect on CRC cell xenograft tumor growth was demonstrated to be mediated through elevated p53-regulated expression of the cell cycle inhibitor p21^Cip1^ and the proapoptotic protein Bax in tumors, as evidenced by qRT-PCR analysis of tumor tissue samples (Figure 7E,F). TUNEL assay results using tumor tissue samples also revealed that apoptosis was further elevated by treatment with the delivered p28-p53-CEABP1 protein in comparison with treatment with p28-p53 (Figure S15).

In summary, fusing the CEABP1 protein to the C-terminus of p28-p53 for specific targeted delivery to CRC cells substantially enhanced the ability of the delivered p53 protein to inhibit CRC cell proliferation and xenograft tumor growth.

### 2.4. Delivery of TCF/LEF TFD DNA by the CPP-Max Protein Suppressed CRC Cell Proliferation and Xenograft Tumor Growth

In 92% of nonhypermutated and 97% of hypermutated CRC cases, mutations occurred in proteins of the Wnt signaling pathway. Some of these mutations happen upstream, such as those in RNF43/ZNRF3; some exist midstream, such as those in APC and Axin; and some occur downstream, such as those in β-catenin or TCF4/TCF3 [4,6]. If we intervene upstream, overactivation of Wnt signaling due to mutations in midstream or downstream components would still not be inhibited. Therefore, we chose to intervene at the most downstream stage of Wnt signaling, which involves the association between the transcription factors TCF/LEF and their target gene promoters.

**Figure 8:**
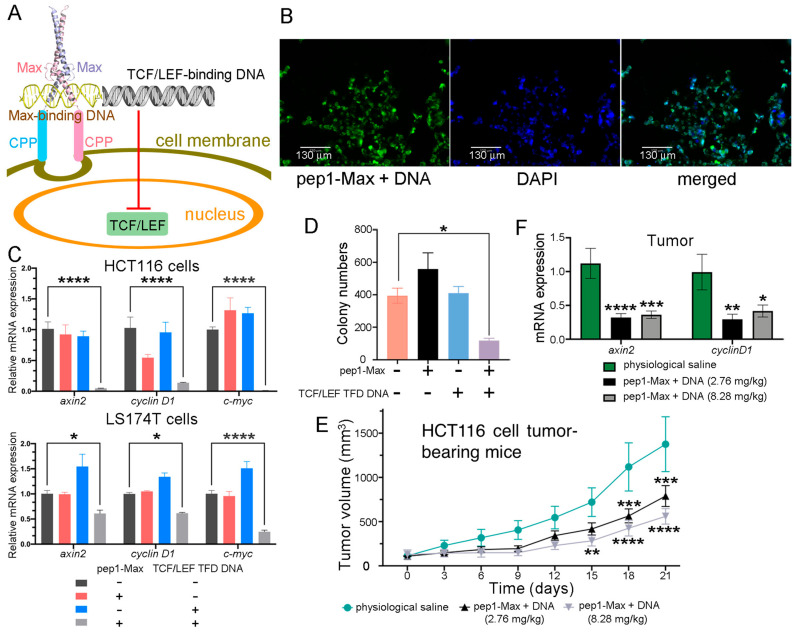
Delivery of TCF/LEF TFD DNA into CRC cells inhibited CRC cell proliferation and xenograft tumor growth. (**A**) The delivery scheme of TCF/LEF TFD DNA. The TCF/LEF-binding DNA was linked with the Max-binding DNA to function as TCF/LEF TFD DNA. The human Max DNA-binding domain was fused with the cell-penetrating peptide pep1, and the fusion protein was employed to deliver the TCF/LEF TFD DNA into CRC cells, which competitively inhibited endogenous TCF/LEF from binding to its target gene promoters and prevented the transcription of Wnt signaling-responsive genes. (**B**) TCF/LEF TFD DNA could be efficiently delivered into LS174T CRC cells and was located in the nuclei. Fluorescence microscopy images of LS174T cells delivered with FAM-labeled TCF/LEF TFD DNA and purified pep1-Max protein are shown. Scale bars: 130 μm. (**C**) Delivery of TCF/LEF TFD DNA suppressed the expression of the Wnt signaling target genes *axin2*, *cyclin D1*, and *c-myc*. TCF/LEF TFD DNA and/or purified pep1-Max protein were delivered into HCT116 or LS174T CRC cells, and qRT-PCR analysis was performed to assess the relative expression levels of *axin2*, *cyclin D1*, and *c-myc* mRNA transcripts. (**D**) Delivery of TCF/LEF TFD DNA by purified pep1-Max protein suppressed HCT116 cell proliferation in the colony formation assay. A quantification of the colony formation assay results is shown. (**E**) Treatment of HCT116 cell xenograft mice via tail vein injection of TCF/LEF TFD DNA and purified pep1-Max protein decelerated tumor growth. Tumor growth curves of HCT116 cell subcutaneous xenograft mice treated with physiological saline (*n* = 6), 2.76 mg of pep1-Max and TCF TFD DNA (molar ratio 3:1) per kg of mice (*n* = 8), or 8.28 mg of pep1-Max and TCF TFD DNA (molar ratio 3:1) per kg of mice (*n* = 7) are shown. Administration started when the tumors grew to 100 mm^3^ in volume. (**F**) Treating mice with TCF/LEF TFD DNA and pep1-Max protein decreased *axin2* and *cyclin D1* mRNA expression in tumors, as revealed by qRT-PCR analysis of tumor samples. Statistical significance is denoted as follows: * *p* < 0.05, ** *p* < 0.01, *** *p* < 0.001, **** *p* < 0.0001.

One of the difficulties in nucleic acid therapeutics is how to deliver nucleic acids into cells. Our approach was to synthesize a short piece (36 base pairs, bp) of double-stranded DNA in which the binding sequence of TCF/LEF and that of a DNA-binding protein were concatenated. Next, the corresponding DNA-binding protein fused with a cell-penetrating peptide was purified and employed to deliver the DNA into CRC cells to function as a TFD to competitively block endogenous TCF/LEF proteins from accessing the promoters of Wnt-responsive genes (Figure 8A). Various DNA-binding proteins, including TetR, the SBD domain of *Streptomyces coelicolor* ScoMcrA [57], and the human Max DNA-binding domain (abbreviated as Max hereafter), and different CPPs, such as TAT, p28, penetratin (pene), and pep1 were tested. Max fused with pep1 and a nuclear localization sequence (abbreviated as pep1-Max hereafter) exhibited the best result in terms of DNA delivery and suppression of CRC cell proliferation, and thus it was selected for subsequent studies. Max dimers preferentially bind to promoter regions containing enhancer box (E-Box) sequences with a 5′-CACGTG-3′ consensus motif [58]. TCF/LEF TFD DNA was synthesized in which the E-Box sequence was linked with the TCF/LEF consensus binding site 5′-AGATCAAAGG-3′, with phosphorothioation modification at the ends of the DNA to reduce degradation by exonucleases [45]. After delivery via the purified pep1-Max protein, the fluorophore FAM-labeled TCF/LEF TFD DNA was indeed internalized into CRC cells and was localized in the nuclei, as indicated by the intense fluorescence signal in nearly all of the cell nuclei (Figure 8B). TCF/LEF TFD DNA without the FAM label was delivered into HCT116 or LS174T CRC cells via purified pep1-Max protein and was indeed found to prevent the transcription of Wnt-responsive genes such as *axin2*, *cyclin D1*, and *c-myc* (Figure 8C). In addition, both the CCK-8 assay (Figure S16) and the colony formation assay (Figure 8D and Figure S17) revealed that the delivered TCF/LEF TFD DNA efficiently suppressed CRC cell proliferation, whereas pep1-Max protein alone or DNA alone had no notable effects on cell growth.

The antitumor effect of TCF/LEF TFD DNA was further investigated in a xenograft tumor mouse model. HCT116 CRC cells were injected subcutaneously into immunodeficient BALB/c nude mice. After the tumors had grown to a certain size, TCF/LEF TFD DNA together with purified pep1-Max protein was systemically injected via the tail vein every 3 days for six consecutive treatments. Compared with the physiological saline control, a significant reduction in tumor volume was observed in the mice treated with TCF/LEF TFD DNA and pep1-Max protein (Figure 8E and Figure S18). At the end of the experiment, a significant decrease in tumor weight was observed for the group of mice receiving 8.28 mg TCF/LEF TFD DNA and pep1-Max per kg of mice compared with the group receiving physiological saline (Figure S19A). Furthermore, the expression of the Wnt signaling target genes *axin2* and *cyclin D1* in tumor tissues was markedly downregulated by the treatment with TCF/LEF TFD DNA and pep1-Max compared with the physiological saline control (Figure 8F). Moreover, TCF/LEF TFD DNA treatment did not affect the body weight (Figure S19B) or spleen weight (Figure S19C) of the mice throughout the experiment, even at higher doses.

Taken together, these results show that delivery of TCF/LEF TFD DNA via the purified pep1-Max protein effectively suppressed CRC cell proliferation and xenograft tumor growth.

### 2.5. Targeting CEA-Expressing CRC Cells with a Designed CEA-Binding Protein Enhanced the Antitumor Effect of TCF/LEF TFD DNA

**Figure 9:**
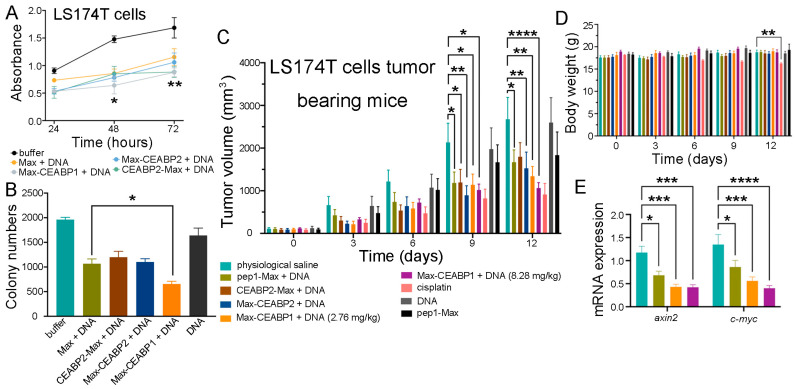
Targeting CEA-expressing CRC cells with a designed CEA-binding protein enhanced the antitumor effect of TCF/LEF TFD DNA. (**A**) Fusion of CEABP1 to the C-terminus of pep1-Max enhanced the suppression of LS174T cell proliferation by the delivered TCF/LEF TFD DNA, which was examined via a CCK-8 assay. The statistics in the figure are for comparison between the pep1-Max-CEABP1 protein plus TCF/LEF TFD DNA group and the pep1-Max protein plus the same DNA group. (**B**) Colony formation assays verified that fusing CEABP1 to pep1-Max caused the delivery of TCF/LEF TFD DNA to inhibit LS174T cell growth more strongly than in the absence of CEABP1. (**C**) Targeting LS174T cell subcutaneous xenograft tumors with CEABP1 enhanced the ability of TCF/LEF TFD DNA to decelerate tumor growth in mice. The tumor growth curves of the following groups receiving different treatments are shown: physiological saline (*n* = 6), 2.76 mg of pep1-Max protein and TCF/LEF TFD DNA per kg of mice (*n* = 6), 2.76 mg of CEABP2-pep1-Max protein and TCF/LEF TFD DNA per kg of mice (*n* = 6), 2.76 mg of pep1-Max-CEABP2 protein and TCF/LEF TFD DNA per kg of mice (*n* = 6), 2.76 mg of pep1-Max-CEABP1 protein and TCF/LEF TFD DNA per kg of mice (*n* = 6), 8.28 mg of pep1-Max-CEABP2 protein and TCF/LEF TFD DNA per kg of mice (*n* = 6), 5 mg cisplatin per kg of mice (*n* = 6), 7.34 mg of pep1-Max protein per kg of mice (*n* = 6), and 0.94 mg of TCF/LEF TFD DNA per kg of mice (*n* = 5). For the above protein and DNA mixtures, the protein-to-DNA molar ratio was 3:1. Administration started when the tumors grew to 100 mm^3^ in volume. (**D**) Fusing CEABP1 or CEABP2 to pep1-Max did not cause any body weight change in the mice during the experiment. (**E**) Specific targeting of CEA-expressing LS174T cells with CEABP1 further decreased the mRNA expression of Wnt-responsive genes in tumors. qRT-PCR analysis of *axin2* and *c-myc* mRNA transcript levels in tumor samples from the indicated groups was performed. Statistical significance is denoted as follows: * *p* < 0.05, ** *p* < 0.01, *** *p* < 0.001, **** *p* < 0.0001.

The designed CEABP1 or CEABP2 protein was fused to the N- or C-terminal end of pep1-Max, and the resulting fusion proteins were expressed, purified, and used to deliver TCF/LEF TFD DNA into LS174T cells. In both the CCK-8 and colony formation assays, significantly greater suppression of CRC cell proliferation was obtained with the purified pep1-Max-CEABP1 protein than with pep1-Max alone. On the other hand, neither pep1-Max-CEABP2 nor CEABP2-pep1-Max consistently showed a stronger ability to inhibit CRC cell growth than pep1-Max (Figure 9A,B and Figure S20).

Next, an in vivo study using immunocompromised athymic nude mice bearing LS174T xenografts was performed, in which TCF/LEF TFD DNA together with purified pep1-Max, CEABP2-pep1-Max, pep1-Max-CEABP2, or pep1-Max-CEABP1 proteins was systemically injected via the tail vein every three days for four successive treatments. LS174T tumor-bearing mice treated with physiological saline, pep1-Max protein alone, or TCF/LEF TFD DNA alone displayed rapid tumor growth, whereas those treated with TCF/LEF TFD DNA in combination with pep1-Max-CEABP1 exhibited more notable decelerated tumor growth than those treated with pep1-Max, CEABP2-pep1-Max, or pep1-Max-CEABP2 (Figure 9C and Figure S21). Additionally, there was no perceivable effect on mouse body weight when mice were treated with TCF/LEF TFD together with CEABP1- or CEABP2-fused pep1-Max (Figure 9D). To evaluate whether specifically targeting CRC cells inhibited Wnt signaling, mRNA from LS174T tumors in different treatment groups was extracted and analyzed via qRT-PCR, and it was found that the use of pep1-Max-CEABP1 to target CEA-expressing CRC cells and deliver TCF/LEF TFD DNA indeed caused higher suppression of Wnt responsive genes such as *axin2* and *c-myc* in the tumor tissue compared to pep1-Max (Figure 9E).

Therefore, the use of a fusion protein of CEABP1 and pep1-Max to specifically target CRC cells further enhanced the suppression of CRC cell proliferation and xenograft tumor growth by TCF/LEF TFD DNA compared with pep1-Max.

## 3. Discussion

p53-based therapeutic strategy presents both unprecedented opportunity and formidable challenges in oncology. Although small-molecule Mdm2 inhibitors such as nutlin have been developed to inhibit p53 ubiquitination by Mdm2 [59], these Mdm2 inhibitors may be effective only for patients with wild-type *TP53* genes and may not work for patients with mutated *TP53*. Ways have been developed to restore the activity of mutated p53 protein, like using arsenic trioxide to bind to allosteric sites on p53 undergoing structural mutations, for example [60]. However, this approach was not effective for the large numbers of DNA-contacting mutants of p53 such as R273H [61,62]. While mutant p53 reactivators such as APR-246 partially restored p53 transcriptional activity in *TP53*-mutant myelodysplastic syndromes, the overall response rates remained modest, with significant interpatient heterogeneity [63]. While current precision oncology frameworks emphasize mutation-specific reactivation of p53, clinical realities reveal staggering heterogeneity resulting from *TP53* mutation spectra. In this work, CRC cell-specific restoration of purified recombinant p53 protein was accomplished via the use of p28 for delivery, a CPP that also inhibits the E3 ubiquitin ligase COP1, and a de novo-designed CEABP1 protein module for targeting. Codelivery of the p28-p14ARF protein significantly prolonged the effective duration of the p53 protein, which not only enhanced antitumor effects but also reduced the dosing frequency in mouse models. Equally noteworthy is that our method could engage erroneous p53 signaling pathways regardless of mutation types.

The integration of cutting-edge computational tools for de novo protein design was pivotal for ensuring CRC-specific targeting in our work. By recognizing CEA, a well-characterized CRC biomarker localized on the cell membrane, the designed CEABP1 protein enhanced the delivery selectivity of p53 protein and TCF/LEF TFD DNA into CRC cells. The increased antitumor activity observed with p28-p53-CEABP1 or pep1-Max-CEABP1 over non-targeted controls p28-p53 or pep1-Max, respectively, underscores the effectiveness of targeted delivery. This finding aligns with the success of antibody-drug conjugates (ADCs) in clinical oncology, and such workflows can be adapted to target other cancer-associated biomarkers for precision cancer therapy. This approach is not guaranteed to be successful, because the binding protein of the tumor biomarker on the cell membrane and the cell penetration peptide may interfere with each other and protein to be delivered may get stuck on the cell membrane and would not be endocytosed into cells.

When we designed the CEA-binding proteins, we took into consideration that the N, A1, and B1 domains of CEA are relatively far away from the cell membrane. After the targeting protein we designed binds to one of these domains of CEA, the cell-penetrating peptide fused with the designed CEA-binding protein might be too distant from the cell membrane to be endocytosed, thereby affecting the efficiency of protein delivery into the CRC cells. As a matter of fact, we have attempted to use the bacterial adhesin protein, which was reported to bind to the N domain of CEA, to fuse with p28-p53 to target CEA-expressing CRC cells. However, it did not work in our functional assays and even weakened the antitumor effect of p28-p53. Therefore, we concluded that the association of adhesin with the N domain of CEA might thwart the membrane entry and protein delivery of p28-p53 protein. In addition, it was reported that CEA was predicted to dimerize [64]. In the dimerized CEA, the A1, B1, and A2 domains from the two CEA protomers were close to each other, which might affect targeting using designed binding proteins. On the other hand, the B3 domain is too close to the cell membrane. The p53 protein we attempted to deliver would occupy some certain spatial volume. In fact, the diameter of the DNA-binding domain is ~43 Å, and the length of the IgG domain of CEA is also ~41 Å. This led us to hypothesize that if we target the A3 or B2 domain of CEA rather than the B3 domain, the fused p28-p53 protein might be in a spatially favorable position to penetrate the cell membrane and be endocytosed into cells. The pLDDT value, which is a measure of the confidence level in Alphafold 3 structure prediction, for the A3 domain of CEA was 93.6874 and that for the B2 domain of CEA was 94.5569 (the full score is 100). Hence, the predicted structures of the A3 and B2 domains of CEA were considered quite reliable.

While previous efforts in the development of Wnt signaling-based anticancer therapeutics have focused on the association between β-catenin and TCF/LEF, our work endeavored to target the subsequent step of recognition between TCF/LEF and its target gene promoters. Our study demonstrated that TCF/LEF TFD DNA conjugated with the pep1-Max-CEABP1 protein could effectively suppress Wnt signaling in CRC cells ex vivo and in vivo, potentially offering a promising strategy to regulate Wnt-driven processes with enhanced specificity and reduced off-target toxicity. Our therapeutic strategy directly targeted the most downstream step of Wnt signaling and could counteract any activating mutation in this pathway. The choice of the pep1-Max-CEABP1 protein as a DNA delivery mediator was crucial, as it not only provided efficient nuclear delivery of TFD DNA but also ensured CRC cell-targeting specificity. This approach overcomes the weakness of traditional TFD DNA delivery methods such as low cellular uptake, improper subcellular localization, and poor targeting specificity for cancer cells. Several noteworthy issues regarding the choice of DNA are as follows. (i) The consensus binding motif of TCF/LEF, 5′-AGATCAAAGG-3′, is relatively long. Therefore, high targeting specificity for TCF/LEF was ensured, and the possibility of off-target binding to other transcription factors was kept low. (ii) The immune response triggered by the DNA-activated cGAS/STING pathway is critically related to the length of the DNA, and the immune response induced by DNA shorter than 45 bp was shown to be weak [65,66]. (iii) The ends of the TFD DNA used were modified by phosphorothioation to prevent excision by exonucleases.

While the present results are encouraging, several challenges remain. The long-term effectiveness and biodistribution of the delivered p53 protein and DNA in vivo require optimization. The potential immunogenicity of synthetic proteins must be reduced to a minimum. There are twenty lysines in the wild-type p53 which are potential ubiquitination sites for at least five E3 ubiquitin ligases reported. An engineered or redesigned p53-like protein in which most or all of the lysines are replaced would be exempt from ubiquitination and degradation and would have a further elongated half-life. A reengineered DNA-binding domain of p53 with higher thermal stability would also benefit from tighter folding and enhanced stability. p53 or TCF/LEF TFD DNA-based therapy could be combined with existing CRC treatments such as chemotherapy or immunotherapy to obtain synergistic effects, and the combination of p53 restoration and modulation of Wnt signaling might also enhance therapeutic efficacy. Our studies could also be extended to the delivery of other tumor suppressor proteins such as Rb or p16INK4a and targeting of other oncogenic transcription factors by TFD DNA via elaborately designed tumor biomarker binding proteins.

## 4. Materials and Methods

### 4.1. Protein Expression and Purification

All of the plasmids were constructed via homologous recombination. Genes encoding various cell-penetrating peptides, including p28, TAT, penetratin (pene), pep1, and the TCF transcription factor decoy DNA, and genes encoding the designed CEA-binding proteins (including CEABP1 and CEABP2) were synthesized by the BGI company. The pMAL-C2x vector was used to express the p28-p53, TAT-p53, pene-p53, pep1-p53, p28-p53-CEABP1, and p28-CEABP2-p53 proteins. The modified pET28a vector with the MBP tag added was used to express the pep1-Max, pep1-Max-CEABP1, pep1-Max-CEABP2, and CEABP2-pep1-Max proteins. The pET28a vector was used to express the GFP-CEABP1 and GFP-CEABP2 proteins. The plasmids were subsequently transformed into *E. coli* BL21(DE3) cells. For protein overexpression, 10 mL of overnight culture was inoculated into 1 L of LB medium supplemented with 100 mg/mL ampicillin or kanamycin. The culture was induced with 0.2 mM IPTG and further incubated at 16 °C for 16 h. The cells were then centrifuged, resuspended in MBP affinity column binding buffer (25 mM Tris–HCl, pH 8.0, 300 mM NaCl, and 2 mM DTT) or Ni^2+^-NTA column binding buffer (25 mM Tris–HCl, pH 8.0, 300 mM NaCl, and 20 mM imidazole), and lysed by a cell homogenizer (JNBio, Guangzhou, China) at 4 °C. After centrifugation (18,000 rpm for 30 min at 4 °C), the supernatant was applied to an MBP affinity column (Smart-Life Sciences, Changzhou, China) and eluted with 20 mM of maltose or applied to a Ni^2+^-NTA affinity column (QIGEN, North Rhine-Westphalia, Germany) and eluted with a 40–500 mM linear concentration gradient of imidazole. The MBP-tagged proteins were cleaved by TEV protease at 4 °C overnight. Finally, the proteins above were further purified by gel filtration chromatography using a Superdex 200 GL 10/300 column (GE Healthcare, Milan, Italy), using phosphate-buffered saline (PBS), pH 7.4, or physiological saline solution. The peak fractions were combined and concentrated to 2 mg/mL. The purified proteins were analyzed via 12% SDS–PAGE and Coomassie blue staining. Protein concentrations were determined via a Bradford protein assay kit (Bio-Rad, Hercules, CA, USA).

### 4.2. Cell Lines and Cell Culture

The cells used in this study included (i) the human colorectal cancer cell line HCT116 (ATCC#CCL-247^TM^), with wild-type p53; (ii) the human colorectal cancer cell line SW480 (ATCC#CCL-228^TM^), with two point mutations of p53 (R273H/P309S); (iii) the human colorectal cancer cell line LS174T (ATCC#CCL-188^TM^), with wild-type p53; (iv) the normal human colonic epithelial cell line NCM460; and (v) the human cervical cancer cell line HeLa (ATCC#CCL-2^TM^). All cells were grown in Dulbecco’s modified Eagle’s medium (DMEM, Corning, Walpole, MA, USA) supplemented with 100 U/mL of penicillin, 100 mg/mL of streptomycin (Gibco, Grand Island, NY, USA), 20 mM of L-glutamine and 10% fetal bovine serum (FBS; Bovogen Biologicals, Melbourne, Australia).

### 4.3. Quantitative Real-Time Reverse Transcription Polymerase Chain Reaction (qRT-PCR)

qRT-PCR was used to quantify the expression of the p53 target genes *cdkn1a* (encoding the p21^Cip1^ protein) and *bax*, as well as the Wnt signal transduction pathway target genes *cyclin D1* and *c-myc* in the HCT116, SW480, LS174T, and HeLa cell lines. The cells were plated in 24-well plates at a density of 5 × 10^5^ cells per well. After 24 h of cell adherence, protein or DNA delivery into the cells was performed via the addition of 20 μg of protein or 1 ng of DNA per well for 6 h. After delivery, 1 mL of fresh complete medium was added, and the cells were further incubated for another 24 or 48 h before RNA extraction and qRT-PCR. Total RNA was isolated via an RNA Extraction Kit (SPARKeasy) according to the manufacturer’s protocol. The RNA was quantified by measuring the UV absorbance at 260 nm. Next, the cDNA was reverse transcribed via a cDNA synthesis kit, PrimeScript™ RT Master Mix Perfect Real Time (Takara, Shiga, Japan). qRT-PCR was performed in a real-time PCR detection instrument with SYBR Green dye (CWBIO). A total of 20 μL of mixture containing 100 ng of cDNA, 2 μM forward and reverse primers each, and 10 μL of 2×MagicSYBR mixture was used for each PCR. The fluorescence signal was recorded at the endpoint of each cycle during the 40 cycles (denaturing at 95 °C for 15 s and annealing at 60 °C for 30 s). *gapdh* was used as an internal control gene for normalization. Relative gene expression was calculated via the 2^−ΔΔCt^ method, which represents the inverse of the amount of mRNA in the initial sample. The sequences of primers used for qRT-PCR are listed below (Table 1). Data were analyzed by qPCRsoft.

### 4.4. CCK-8 Cell Proliferation Assay

Colorectal cancer cell proliferation was examined via the Cell Counting Kit-8 (CCK-8) assay (Meilunbio, Dalian, China). The cancer cells were plated in 24-well plates at a density of 5 × 10^5^ cells per well. After 24 h of cell adherence, protein or DNA delivery into cells was performed via the addition of 20 μg of protein or 1 ng of DNA per well for 6 h. Afterwards, the cells were detached with 0.25% EDTA-treated trypsin and seeded into 96-well plates at a density of 3 × 10^3^ cells per well. After 24, 48, or 72 h, the old medium was discarded, and 0.1 mL of new medium without FBS but containing 0.01 mL of CCK8 solution was added to each well and incubated for 0.5–4 h. The absorbance was determined at a wavelength of 450 nm via a microplate reader.

### 4.5. Colony Formation Assay

The cells were treated with purified p53 protein or DNA for 48 h. Then, the cells were detached with 0.25% EDTA-treated trypsin, seeded into 6-well plates at a low density (~1000 cells per well), and incubated for 2 weeks. The plates were then washed with PBS, fixed in 4% paraformaldehyde for 20 min, and then stained with 0.005% crystal violet. The images of all the wells were scanned and analyzed.

### 4.6. Ethynyl Deoxyuridine (EdU) Incorporation Assay

The cell proliferation rate was assessed via the EdU assay according to the manufacturer’s instructions. Cells were seeded in 96-well plates, treated with various concentrations of purified p28-p53 protein for 72 h, and incubated with EdU-labeling medium at 37 °C for 4 h. The cells were then fixed with 4% paraformaldehyde for 15 min and permeabilized with 0.3% Triton X-100 for an additional 15 min. Following PBS buffer washes, the cells were incubated with click reagent (1×) solution for 30 min in the dark at room temperature. Nuclei were stained with Hoechst 33342 solution (1×) and observed under a fluorescence microscope (Echo Revolve, San Diego, CA, USA).

### 4.7. Terminal Deoxynucleotidyl Transferase-Mediated dUTP-Biotin Nick End Labeling (TUNEL) Assay

Apoptotic cells were measured with a One Step TUNEL Apoptosis Assay Kit (Beyotime, Shanghai, China) according to the manufacturer’s protocol. Tumors were extracted and fixed in formalin, embedded in paraffin, and sectioned at a thickness of 5 μm. TUNEL-positive cells had pyknotic nuclei with red fluorescence, indicating apoptosis. Images of the sections were taken with a fluorescence microscope (Echo).

### 4.8. Cell Cycle Analysis

The cell cycle was analyzed with a Cell Cycle and Apoptosis Analysis Kit (Beyotime). For cell cycle analysis, samples of 1 × 10^6^ cells were fixed and permeabilized via the addition of 1 mL of ice-cold 70% ethanol for 15 min on ice. After washing, the cells were resuspended in 125 μL of 1.12% (*w*/*v*) sodium citrate containing 0.2 mg/mL RNase (Beyotime) and incubated at 37 °C for 15 min. Next, 125 μL of 1.12% (*w*/*v*) sodium citrate containing 50 μg/mL propidium iodide (Beyotime) was added to the cells. Following treatment for 30 min at room temperature in the dark, the cells were stored at 4 °C until analyzed via flow cytometry (FACSCalibur, BD Biosciences, Franklin Lakes, NJ, USA). Cell cycle analysis was performed via ModFit LT software v 5.0.

### 4.9. Xenograft Tumor Model and Administration of the p28-p53 Protein or TCF/LEF TFD DNA and pep1-Max Protein in the Presence or Absence of CEA-Binding Protein

HCT116 or LS174T cells were inoculated subcutaneously into the flanks of female BALB/c athymic nude mice (2 × 10^6^ cells per 200 μL or 1 × 10^7^ per 200 μL). Seven to ten days later, the animals were grouped by randomizing the tumor sizes (approximately 70–120 mm^3^). Five to six animals were used per group. Generally speaking, meditation of p53 protein or TFD DNA was administered starting from a tumor volume of 100 mm^3^ unless otherwise stated. The purified protein or transcription factor decoy DNA plus the Max protein were injected via the tail vein once every 3 days, with 100 μL of total volume per injection. The group of mice receiving physiological saline solution injections was used as a negative control, whereas the group receiving cisplatin injection was used as a positive control. Moreover, the lengths and widths of the tumors were measured every 3 days with a Vernier caliper. The tumor volumes were calculated via the following formula: volume = (length × width^2^)/2 for each animal.

### 4.10. De Novo Design of the CEA-Binding Proteins CEABP1 and CEABP2

The RFdiffusion method was used to construct the scaffolds of the designed CEA-binding proteins, including CEABP1 and CEABP2. By introducing noise into the initially randomly arranged amino acid residues and considering the CEA structure, the process iteratively reduces the noise while simulating protein dynamics in a physiological environment. This results in a scaffold structure that optimally folds and spatially complements the specific binding sites of CEA. During this process, energy functions and geometric constraints are applied to optimize the scaffold, avoiding local potential traps. Each iteration step evaluates and corrects the scaffold on the basis of the structural features of the target protein, making it more compatible with the binding interface or functional regions on the CEA surface. Ultimately, designs with high stability and optimal complementarity are selected.

The ProteinMPNN method was employed to design the amino acid sequences of the designed CEA-binding proteins. During this procedure, the graph neural networks and a message-passing mechanism were utilized to integrate spatial and energy information. This method selects the most appropriate amino acid type for each residue position, ensuring high compatibility between the sequence and scaffold while maintaining excellent folding potential. In the design of CEA-binding proteins, the ProteinMPNN focuses particularly on optimizing the hydrophobic cores, the hydrogen bond networks, and the salt bridges at the protein-protein binding interfaces, significantly increasing the binding affinity and specificity. Through a “scaffold-sequence” two-step approach, a set of candidate CEA-binding proteins with high binding affinity, thermal stability, and expression capacity were designed by inputting parameters such as the three-dimensional structure of the CEA, hotspot residues, and noise reduction scale. The RFdiffusion technology addresses the challenges of spatial geometric constraints and functional residue accommodation, whereas the ProteinMPNN method ensures stable folding of the residue combinations and high-selectivity binding. The synergy between these two technologies enhances the functionality and expressibility of the designed proteins.

Finally, AlphaFold3 and Rosetta were used to exclude poorly folded candidate sequences. Protein-target binding was then evaluated to reduce the false positive rate. AlphaFold3 was used to predict the protein complex structure, focusing on the predicted alignment error (PAE) and root mean square deviation (RMSD) metrics to assess the stability of the interfaces between CEA and the designed CEA-binding proteins. Rosetta was employed to quantify the free energy of the binding interface, analyze the hydrogen bonds and spatial complementarity, and address potential issues through iterative optimization. Using a custom weight function that combines AlphaFold3 evaluation metrics with Rosetta’s energy functions, several sequences were selected through multiple rounds of screening and redesign. Molecular dynamics simulations were performed to observe the dynamic interaction between the designed CEA-binding proteins and the target protein, evaluating the stability of the binding interface and selecting structures with greater dynamic stability and tighter specific binding. Through multiple rounds of evaluation and selection among 10,000 to 100,000 candidate designed sequences in silico, 5 to 10 designed sequences of CEA-binding proteins were selected for experimental validation.

### 4.11. Molecular Dynamics (MD) Simulation

AlphaFold3 was used to predict complex structures, and topologies for MD simulations were generated with pdb4amber and tleap, tools from the AMBER suite. MD simulations were carried out via AMBER, which employs the TIP3P water model and the ff14SB force field for solvent and protein interactions. Two-step energy minimization was performed, first focusing on the solvent and then the whole system. After minimization, the system was gradually heated from 0 K to 300 K over 50 pecoseconds (ps). Equilibration was performed under NPT conditions at 300 K and 1 bar for 50 ns for density stabilization. The equilibrated structures were used for production simulations lasting at least 400 ns to generate structural data. SHAKE was applied after minimization to constrain hydrogen bonds, and PME was used for long-range electrostatics. Trajectory analysis was performed using Cpptraj and MDAnalysis, and visualizations were created via PyMOL.

### 4.12. Immunofluorescence Staining

The cells that were incubated with GFP-tagged CEA-binding proteins (CEABP1 or CEABP2) were fixed with 4% paraformaldehyde at room temperature for 15 min. The samples were further incubated with PBS blocking buffer (containing 2% BSA and 2% nonfat milk) at room temperature for 30 min. Afterwards, the samples were incubated with primary antibody overnight at 4 °C, washed with PBS, and incubated with goat anti-rat-Alexa Fluor 647 (Yeasen, Shanghai, China) in blocking buffer (1:1000 dilution) at room temperature for 60 min. The stained samples were washed with PBS, and the nuclei were stained with Hoechst 33342 (MA0126, Meilunbio, Dalian, China; 1:100 dilution in PBS). The samples were mounted on slides with Fluoromount-G™ mounting medium (Yeason).

### 4.13. Hematoxylin & Eosin (H&E) Staining

The paraffin-embedded slides were first deparaffinized with xylene, followed by a series of grades of ethanol, and finally with water. The tissues on the slides were then incubated with a hematoxylin solution for one minute and washed with tap water until the water was clear. Next, the tissues were counterstained in an eosin solution for 15 s. After counterstaining, the tissue slides were immediately transferred to 95% ethanol and further dehydrated with 100% ethanol and xylene. Finally, the slides were mounted with neutral balsam mounting medium and air-dried before imaging.

### 4.14. Antibodies

The antibodies for CEA (Proteintech Cat#: 10421-1-AP) and Alexa Fluor 594-conjugated goat anti-rabbit IgG (H+ L) (ABclonal Cat#: AS039) were used in this study.

### 4.15. Statistical Analysis

All of the graphs were prepared via GraphPad Prism 9 software, and statistical analysis was also carried out via GraphPad Prism 9 software to perform one-way ANOVA, two-sided *t* tests, or two-way ANOVA. The error bars indicate the standard error of the mean (SEM). A *p* value < 0.05 is considered statistically significant, where all statistically significant values shown in the figures are indicated as follows: * *p* < 0.05, ** *p* < 0.01, *** *p* < 0.001, and **** *p* < 0.0001.

## Figures and Tables

**Table 1:** Primers sequences for qRT-PCR.

Gene	Forward Primer	Reverse Primer
*gapdh*	ATGTGGCCGAGGACTTTGATT	AGTGGGGTGGCTTTTAGGATG
*cdkn1a*	CTTTCTGGCCGTCAGGAACA	CTTCTATGCCAGAGCTCAACATGT
*bax*	TCAGGATGCGTCCACCAAGAAG	TGTGTCCACGGCGGCAATCATC
*axin2*	AGCCAAAGCGATCTACAAAAG	AAGTCAAAAACATCTGGTAGGCA
*cyclin D1*	CAGAGTGATCAAGTGTGACCC	CGTCGGTGGGTGTGCAAGC
*c-myc*	CTCCTACGTTGCGGTCACAC	CGGGTCGCAGATGAAACTCT

## Data Availability

The cell lines that support the findings of this study are openly available in https://www.atcc.org (accessed on 4 June 2021 for HCT116 cell, and 9 April 2023 for LS174T cell).

## Supplementary Materials

The following supporting information can be downloaded at: https://www.mdpi.com/article/10.3390/ijms26209846/s1.

## References

[1] Bray F., Laversanne M., Sung H., Ferlay J., Siegel R.L., Soerjomataram I., Jemal A. (2024). Global cancer statistics 2022: GLOBOCAN estimates of incidence and mortality worldwide for 36 cancers in 185 countries. CA Cancer J. Clin..

[2] Eng C., Yoshino T., Ruíz-García E., Mostafa N., Cann C.G., O’Brian B., Benny A., Perez R.O., Cremolini C. (2024). Colorectal cancer. Lancet.

[3] Jemal A., Bray F., Center M.M., Ferlay J., Ward E., Forman D. (2011). Global cancer statistics. CA Cancer J. Clin..

[4] Cancer Genome Atlas Network (2012). Comprehensive molecular characterization of human colon and rectal cancer. Nature.

[5] Nguyen L.H., Goel A., Chung D.C. (2020). Pathways of colorectal carcinogenesis. Gastroenterology.

[6] Li J., Ma X., Chakravarti D., Shalapour S., DePinho R.A. (2021). Genetic and biological hallmarks of colorectal cancer. Genes Dev..

[7] Dekker E., Tanis P.J., Vleugels J.L.A., Kasi P.M., Wallace M.B. (2019). Colorectal cancer. Lancet.

[8] Baker S.J., Preisinger A.C., Jessup J.M., Paraskeva C., Markowitz S., Willson J.K., Hamilton S., Vogelstein B. (1990). p53 gene mutations occur in combination with 17p allelic deletions as late events in colorectal tumorigenesis. Cancer Res..

[9] Kinzler K.W., Vogelstein B. (1996). Lessons from hereditary colorectal cancer. Cell.

[10] Lane D.P. (1992). p53, guardian of the genome. Nature.

[11] Harris S.L., Levine A.J. (2005). The p53 pathway: Positive and negative feedback loops. Oncogene.

[12] Levine A.J., Oren M. (2009). The first 30 years of p53: Growing ever more complex. Nat. Rev. Cancer.

[13] Liu Y., Su Z., Tavana O., Gu W. (2024). Understanding the complexity of p53 in a new era of tumor suppression. Cancer Cell.

[14] el-Deiry W.S., Tokino T., Velculescu V.E., Levy D.B., Parsons R., Trent J.M., Lin D., Mercer W.E., Kinzler K.W., Vogelstein B. (1993). WAF1, a potential mediator of p53 tumor suppression. Cell.

[15] Harper J.W., Adami G.R., Wei N., Keyomarsi K., Elledge S.J. (1993). The p21 Cdk-interacting protein Cip1 is a potent inhibitor of G1 cyclin-dependent kinases. Cell.

[16] Miyashita T., Reed J.C. (1995). Tumor suppressor p53 is a direct transcriptional activator of the human *bax* gene. Cell.

[17] Oda E., Ohki R., Murasawa H., Nemoto J., Shibue T., Yamashita T., Tokino T., Taniguchi T., Tanaka N. (2000). Noxa, a BH3-only member of the Bcl-2 family and candidate mediator of p53-induced apoptosis. Science.

[18] Nakano K., Vousden K.H. (2001). *PUMA*, a novel proapoptotic gene, is induced by p53. Mol. Cell.

[19] Yu J., Zhang L., Hwang P.M., Kinzler K.W., Vogelstein B. (2001). PUMA induces the rapid apoptosis of colorectal cancer cells. Mol. Cell.

[20] Haupt Y., Maya R., Kazaz A., Oren M. (1997). Mdm2 promotes the rapid degradation of p53. Nature.

[21] Kubbutat M.H., Jones S.N., Vousden K.H. (1997). Regulation of p53 stability by Mdm2. Nature.

[22] Leng R.P., Lin Y., Ma W., Wu H., Lemmers B., Chung S., Parant J.M., Lozano G., Hakem R., Benchimol S. (2003). Pirh2, a p53-induced ubiquitin-protein ligase, promotes p53 degradation. Cell.

[23] Dornan D., Wertz I., Shimizu H., Arnott D., Frantz G.D., Dowd P., O’Rourke K., Koeppen H., Dixit V.M. (2004). The ubiquitin ligase COP1 is a critical negative regulator of p53. Nature.

[24] Chen D., Kon N., Li M., Zhang W., Qin J., Gu W. (2005). ARF-BP1/Mule is a critical mediator of the ARF tumor suppressor. Cell.

[25] Kamijo T., Zindy F., Roussel M.F., Quelle D.E., Downing J.R., Ashmun R.A., Grosveld G., Sherr C.J. (1997). Tumor suppression at the mouse INK4a locus mediated by the alternative reading frame product p19ARF. Cell.

[26] Pomerantz J., Schreiber-Agus N., Liégeois N.J., Silverman A., Alland L., Chin L., Potes J., Chen K., Orlow I., Lee H.W. (1998). The Ink4a tumor suppressor gene product, p19Arf, interacts with MDM2 and neutralizes MDM2’s inhibition of p53. Cell.

[27] Zhang Y., Xiong Y., Yarbrough W.G. (1998). ARF promotes MDM2 degradation and stabilizes p53: ARF-INK4a locus deletion impairs both the Rb and p53 tumor suppression pathways. Cell.

[28] Weber J.D., Taylor L.J., Roussel M.F., Sherr C.J., Bar-Sagi D. (1999). Nucleolar Arf sequesters Mdm2 and activates p53. Nat. Cell Biol..

[29] Tao W., Levine A.J. (1999). p19ARF stabilizes p53 by blocking nucleo-cytoplasmic shuttling of Mdm2. Proc. Natl. Acad. Sci. USA.

[30] Vogelstein B., Fearon E.R., Hamilton S.R., Kern S.E., Preisinger A.C., Leppert M., Nakamura Y., White R., Smits A.M., Bos J.L. (1998). Genetic alterations during colorectal-tumor development. N. Engl. J. Med..

[31] Behrens J., von Kries J.P., Kühl M., Bruhn L., Wedlich D., Grosschedl R., Birchmeier W. (1996). Functional interaction of beta-catenin with the transcription factor LEF-1. Nature.

[32] Nusse R., Clevers H. (2017). Wnt/beta-catenin signaling, disease, and emerging therapeutic modalities. Cell.

[33] Rim E.Y., Clevers H., Nusse R. (2022). The Wnt Pathway: From signaling mechanisms to synthetic modulators. Annu. Rev. Biochem..

[34] Takenobu T., Tomizawa K., Matsushita M., Li S.T., Moriwaki A., Lu Y.F., Matsui H. (2002). Development of p53 protein transduction therapy using membrane-permeable peptides and the application to oral cancer cells. Mol. Cancer Ther..

[35] Ryu J., Lee H.J., Kim K.A., Lee J.Y., Lee K.S., Park J., Choi S.Y. (2004). Intracellular delivery of p53 fused to the basic domain of HIV-1 Tat. Mol. Cells.

[36] Michiue H., Tomizawa K., Wei F.Y., Matsushita M., Lu Y.F., Ichikawa T., Tamiya T., Date I., Matsui H. (2005). The NH2 terminus of influenza virus hemagglutinin-2 subunit peptides enhances the antitumor potency of polyarginine-mediated p53 protein transduction. J. Biol. Chem..

[37] Hitsuda T., Michiue H., Kitamatsu M., Fujimura A., Wang F., Yamamoto T., Han X.J., Tazawa H., Uneda A., Ohmori I. (2012). A protein transduction method using oligo-arginine (3R) for the delivery of transcription factors into cell nuclei. Biomaterials.

[38] Guidotti G., Brambilla L., Rossi D. (2017). Cell-penetrating peptides: From basic research to clinics. Trends Pharmacol. Sci..

[39] Zhao M., Liu Y., Hsieh R.S., Wang N., Tai W., Joo K.I., Wang P., Gu Z., Tang Y. (2014). Clickable protein nanocapsules for targeted delivery of recombinant p53 protein. J. Am. Chem. Soc..

[40] Yang Z., Lee M.M.M., Chan M.K. (2021). Efficient intracellular delivery of p53 protein by engineered protein crystals restores tumor suppressing function in vivo. Biomaterials.

[41] Yang Z., Sun J.K., Lee M.M., Chan M.K. (2022). Restoration of p53 activity via intracellular protein delivery sensitizes triple negative breast cancer to anti-PD-1 immunotherapy. J. Immunother. Cancer.

[42] Zhao H., Ming T., Tang S., Ren S., Yang H., Liu M., Tao Q., Xu H. (2022). Wnt signaling in colorectal cancer: Pathogenic role and therapeutic target. Mol. Cancer.

[43] Jin J.O., Kim G., Hwang J., Han K.H., Kwak M., Lee P.C.W. (2020). Nucleic acid nanotechnology for cancer treatment. Biochim. Biophys. Acta Rev. Cancer.

[44] Hammond S.M., Aartsma-Rus A., Alves S., Borgos S.E., Buijsen R.A.M., Collin R.W.J., Covello G., Denti M.A., Desviat L.R., Echevarría L. (2021). Delivery of oligonucleotide-based therapeutics: Challenges and opportunities. EMBO Mol. Med..

[45] Belgrad J., Fakih H.H., Khvorova A. (2024). Nucleic acid therapeutics: Successes, milestones, and upcoming innovation. Nucleic Acid Ther..

[46] Mann M.J., Dzau V.J. (2000). Therapeutic applications of transcription factor decoy oligonucleotides. J. Clin. Investig..

[47] Hecker M., Wagner A.H. (2017). Transcription factor decoy technology: A therapeutic update. Biochem. Pharmacol..

[48] Johari B., Moradi M. (2022). Application of transcription factor decoy oligodeoxynucleotides (ODNs) for cancer therapy. Methods Mol. Biol..

[49] Hammarström S. (1999). The carcinoembryonic antigen (CEA) family: Structures, suggested functions and expression in normal and malignant tissues. Semin. Cancer Biol..

[50] Beauchemin N., Arabzadeh A. (2013). Carcinoembryonic antigen-related cell adhesion molecules (CEACAMs) in cancer progression and metastasis. Cancer Metastasis Rev..

[51] Yamada T., Fialho A.M., Punj V., Bratescu L., Gupta T.K., Chakrabarty A.M. (2005). Internalization of bacterial redox protein azurin in mammalian cells: Entry domain and specificity. Cell. Microbiol..

[52] Yamada T., Mehta R.R., Lekmine F., Christov K., King M.L., Majumdar D., Shilkaitis A., Green A., Bratescu L., Beattie C.W. (2009). A peptide fragment of azurin induces a p53-mediated cell cycle arrest in human breast cancer cells. Mol. Cancer Ther..

[53] Yamada T., Christov K., Shilkaitis A., Bratescu L., Green A., Santini S., Bizzarri A.R., Cannistraro S., Das Gupta T.K., Beattie C.W. (2013). p28, a first in class peptide inhibitor of cop1 binding to p53. Br. J. Cancer.

[54] Taylor B.N., Mehta R.R., Yamada T., Lekmine F., Christov K., Chakrabarty A.M., Green A., Bratescu L., Shilkaitis A., Beattie C.W. (2009). Noncationic peptides obtained from azurin preferentially enter cancer cells. Cancer Res..

[55] Watson J.L., Juergens D., Bennett N.R., Trippe B.L., Yim J., Eisenach H.E., Ahern W., Borst A.J., Ragotte R.J., Milles L.F. (2023). De novo design of protein structure and function with RFdiffusion. Nature.

[56] Dauparas J., Anishchenko I., Bennett N., Bai H., Ragotte R.J., Milles L.F., Wicky B.I.M., Courbet A., de Haas R.J., Bethel N. (2022). Robust deep learning-based protein sequence design using ProteinMPNN. Science.

[57] Liu G., Fu W., Zhang Z., He Y., Yu H., Wang Y., Wang X., Zhao Y.L., Deng Z., Wu G. (2018). Structural basis for the recognition of sulfur in phosphorothioated DNA. Nat. Commun..

[58] Ferré-D’Amaré A.R., Prendergast G.C., Ziff E.B., Burley S.K. (1993). Recognition by Max of its cognate DNA through a dimeric b/HLH/Z domain. Nature.

[59] Vassilev L.T., Vu B.T., Graves B., Carvajal D., Podlaski F., Filipovic Z., Kong N., Kammlott U., Lukacs C., Klein C. (2004). In vivo activation of the p53 pathway by small-molecule antagonists of MDM2. Science.

[60] Chen S., Wu J.L., Liang Y., Tang Y.G., Song H.X., Wu L.L., Xing Y.F., Yan N., Li Y.T., Wang Z.Y. (2021). Arsenic trioxide rescues structural p53 mutations through a cryptic allosteric site. Cancer Cell.

[61] Cho Y., Gorina S., Jeffrey P.D., Pavletich N.P. (1994). Crystal structure of a p53 tumor suppressor-DNA complex: Understanding tumorigenic mutations. Science.

[62] Song H., Wu J., Tang Y., Dai Y., Xiang X., Li Y., Wu L., Wu J., Liang Y., Xing Y. (2023). Diverse rescue potencies of p53 mutations to ATO are predetermined by intrinsic mutational properties. Sci. Transl. Med..

[63] Sallman D.A., DeZern A.E., Garcia-Manero G., Steensma D.P., Roboz G.J., Sekeres M.A., Cluzeau T., Sweet K.L., McLemore A., McGraw K.L. (2021). Eprenetapopt (APR-246) and azacitidine in *TP53*-mutant myelodysplastic syndromes. J. Clin. Oncol..

[64] Shabo I., Nordling E., Abraham-Nordling M. (2005). Artificial intelligence prediction of carcinoembryonic antigen structure and interactions relevant for colorectal cancer. Biochem. Biophys. Rep..

[65] Luecke S., Holleufer A., Christensen M.H., Jønsson K.L., Boni G.A., Sørensen L.K., Johannsen M., Jakobsen M.R., Hartmann R., Paludan S.R. (2017). cGAS is activated by DNA in a length-dependent manner. EMBO Rep..

[66] Zhou W., Whiteley A.T., de Oliveira Mann C.C., Morehouse B.R., Nowak R.P., Fischer E.S., Gray N.S., Mekalanos J.J., Kranzusch P.J. (2018). Structure of the human cGAS-DNA complex reveals enhanced control of immune surveillance. Cell.

