# Supplementary material for: Targeted Inhibition of Colorectal Carcinoma Using a Designed CEA-Binding Protein to Deliver p53 Protein and TCF/LEF Transcription Factor Decoy DNA
Source: Int J Mol Sci. 2025 Oct 10;26(20):9846. doi: 10.3390/ijms26209846 (PMC12564183; doi:10.3390/ijms26209846)
Supplement: Supplementary file 1 [file ijms-26-09846-s001.zip › ijms-3856792-supplementary.pdf]

SUPPLEMENTARY INFORMATION

SUPPLEMENTARY FIGURES

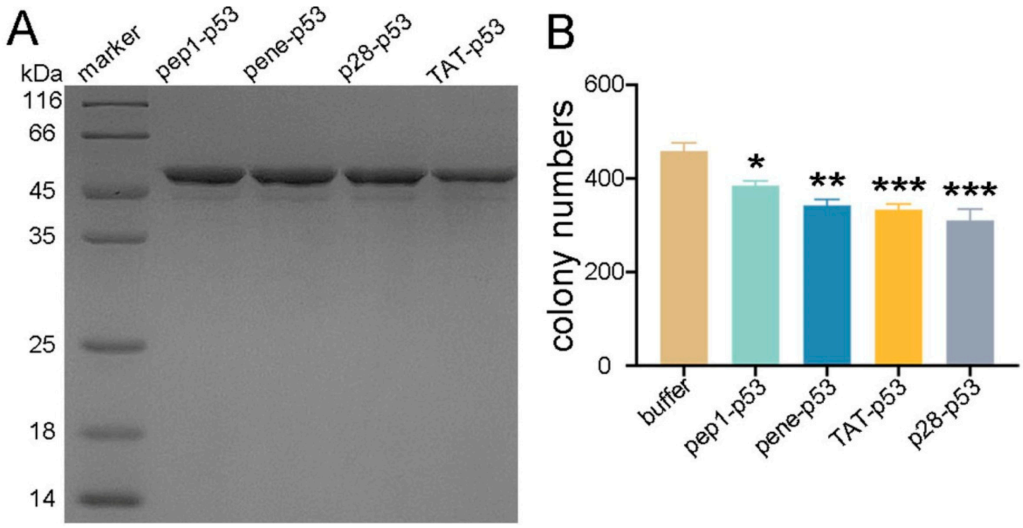

Figure S1: Purified full length human p53 protein fused with the p28 cell-penetrating peptide displayed the highest level of inhibition on HCT116 cells proliferation in the colony formation assay. (A) Purification of the full-length human p53 protein fused with various cell-penetrating peptides: pep1, penetratin (pene), p28, and TAT. (B) Purified p28-p53 protein exhibited the strongest inhibition of cell proliferation when delivered into HCT116 cells, as shown by the colony formation assay.

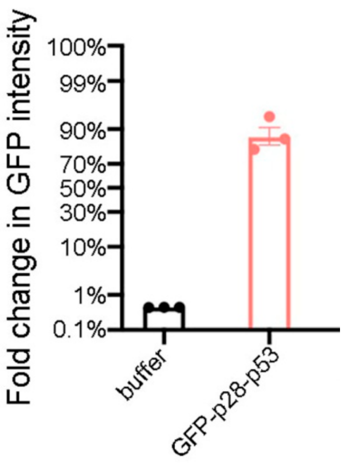

Figure S2: Purified GFP-tagged p53 protein could be highly efficiently delivered into HCT116 cells via the cell penetrating peptide p28, as suggested by the fold change in GFP fluorescence intensity after protein delivery.

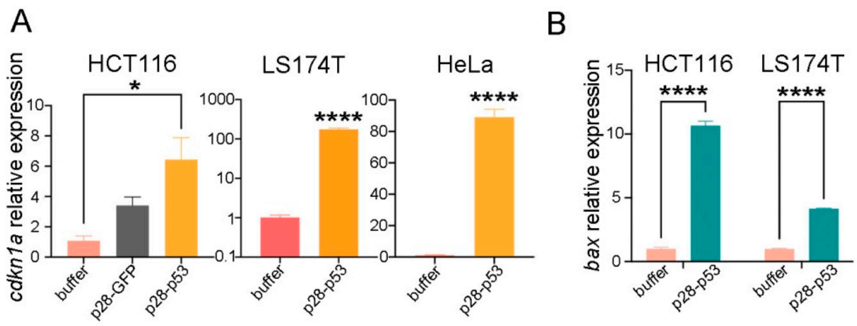

Figure S3: Delivery of the p28-p53 protein into cancer cells enhanced the transcription of p53 target genes *cdkn1a* and *bax*. (A) Delivery of the p28-p53 protein effectively increased *cdkn1a* transcript levels in HCT116, LS174T, and HeLa cells, as revealed by qRT–PCR analysis. (B) Delivery of the p28-p53 protein also increased the transcript level of *bax* in HCT116 and LS174T cells, as shown by qRT–PCR analysis.

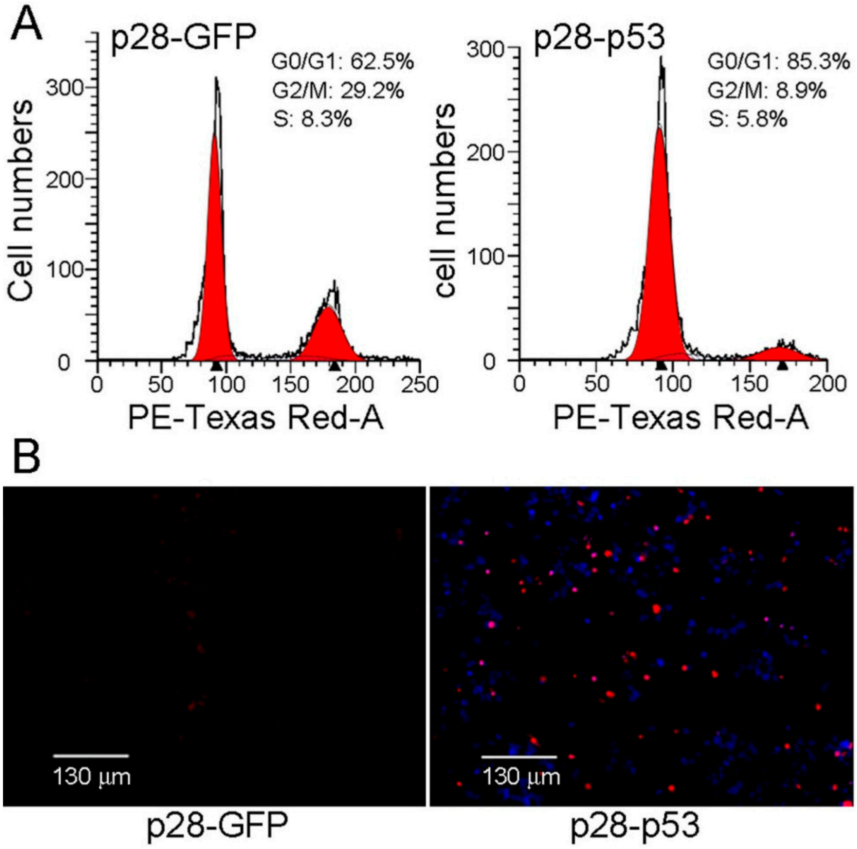

Figure S4: Delivery of the p28-p53 protein into HCT116 CRC cells caused cell cycle arrest and apoptosis. (A) Delivery of the p28-p53 protein caused cell cycle arrest at the G0/G1 phase in HCT116 cells. The cell cycle distribution was determined via flow cytometry. (B) After delivery, p28-p53 protein triggered apoptosis in HCT116 cells, as revealed by the TUNEL assay. The red dots represented apoptotic cells.

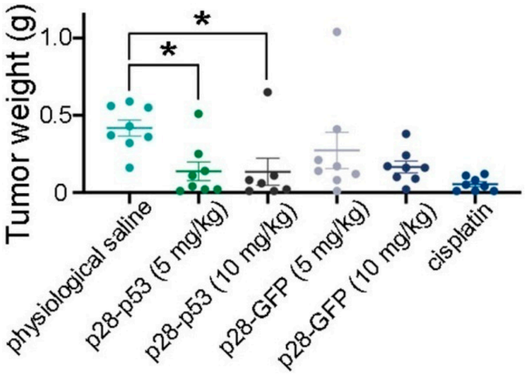

Figure S5: The weight of HCT116 cells xenograft tumors at the experimental endpoint, corresponding to Figure 2D.

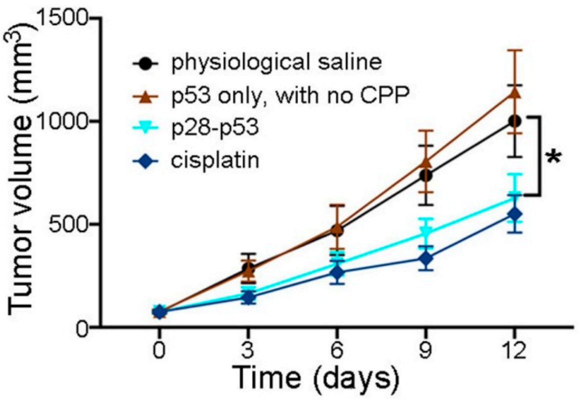

Figure S6: Purified p28-p53 protein most effectively restrained the growth of HCT116 cells subcutaneous xenograft tumors in mice. The tumor growth curves of the following groups receiving different protein delivery treatments are shown: physiological saline solution (n = 7), 10 mg p53 without CPP (n = 8) per kg of mice, 10 mg p28-p53 per kg of mice (n = 8), and 5 mg cisplatin per kg of mice (n = 8).

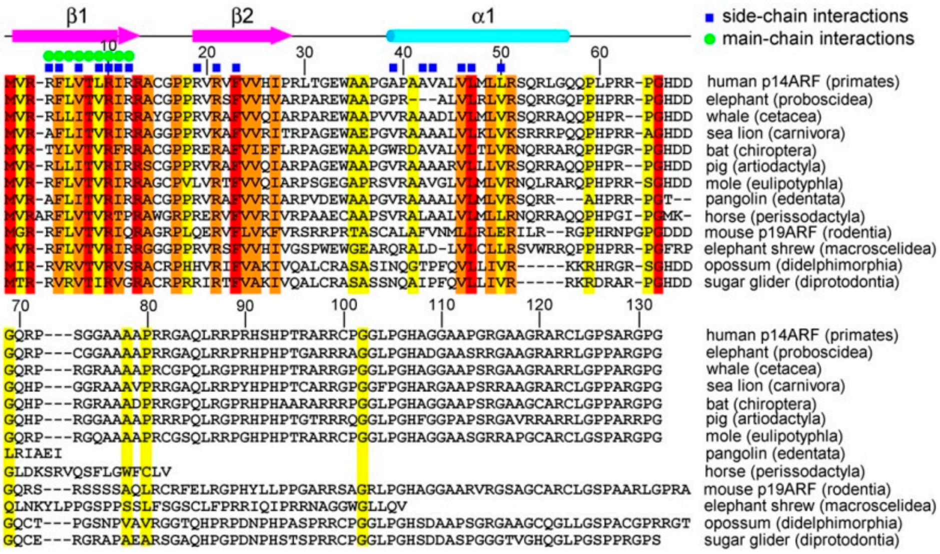

Figure S7: The N-terminal 63 residues of p14ARF are highly conserved. Multiple sequence alignments were performed for representative p14ARF orthologs from human, African savanna elephant (*Loxodonta africana*, accession number: XP_064146496), gray whale (*Eschrichtius robustus*, XP_068408523), Steller sea lion (*Eumetopias jubatus*, XP_027970991), bat (*Myotis myotis*, KAF6314295), pig (*Sus scrofa*, CAD53376), Iberian mole (*Talpa occidentalis*, XP_054545170), Chinese pangolin (*Manis pentadactyla*, KAI5158232), Przewalski's horse (*Equus przewalskii*, XP_008508663), mouse p19ARF, cape elephant shrew (*Elephantulus edwardii*, XP_006881402), gray short-tailed opossum (*Monodelphis domestica*, NP_001028145), and sugar glider (*Petaurus breviceps papuanus*, XP_068943629).

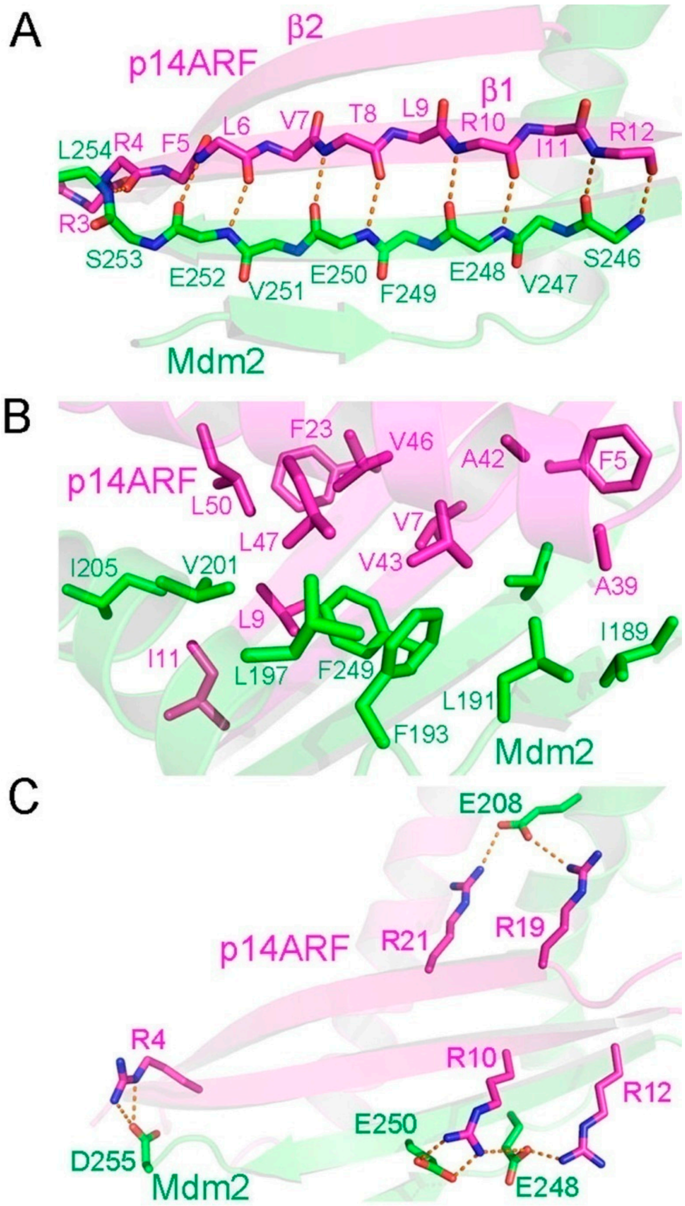

Figure S8: p14ARF interacts with Mdm2 through main-chain hydrogen bonding interactions, as well as side-chain hydrophobic and electrostatic interactions. (A) Main-chain hydrogen bonding interactions between p14ARF and Mdm2. (B) Side-chain hydrophobic interactions between p14ARF and Mdm2. (C) Side-chain electrostatic interactions between p14ARF and Mdm2.

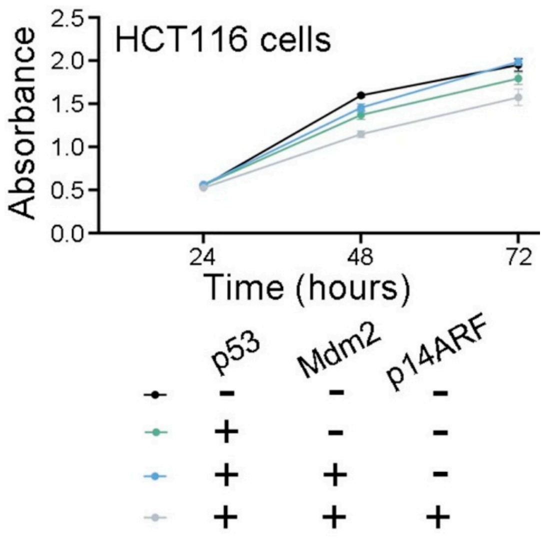

Figure S9: The delivered p14ARF protein enhanced p53's inhibitory effect on CRC cell proliferation, as shown by the CCK-8 assay. Purified p28-p14ARF and p28-p53 proteins were delivered while an Mdm2-encoding plasmid was transfected into HCT116 cells.

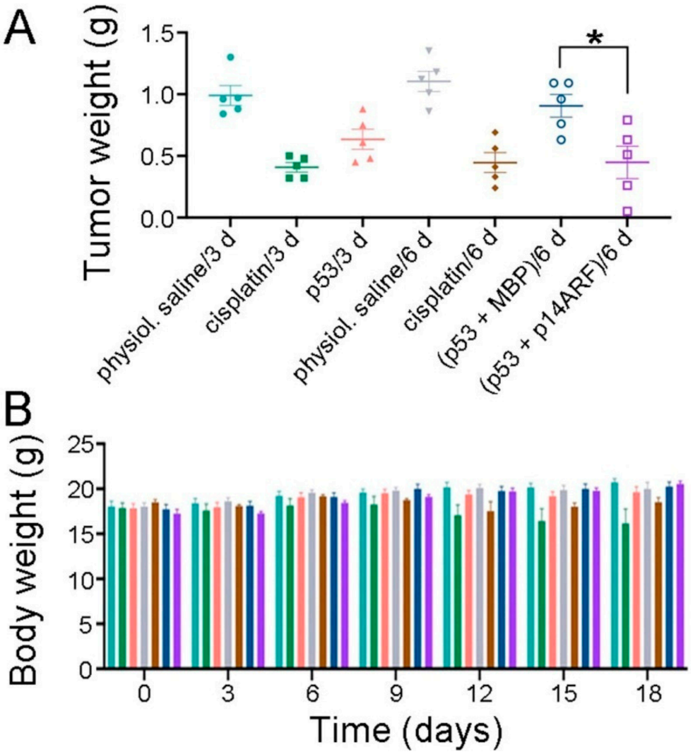

Figure S10: The tumor weights at the experimental endpoint and mice body weights in the HCT116 cells xenograft tumor experiment with administration of purified p28-p53 and p28-p14ARF proteins. (A) The HCT116 cell xenograft tumor weights at the experimental endpoint corresponding to Figures 4A and 4B. The color scheme for the different groups was the same as that described in Figure 4A. (B) Treatment with purified p28-p53 and p28-p14ARF proteins did not cause body weight changes in the mice during the experiment, corresponding to Figure 4A. The color scheme for the different groups was the same as that described in Figure 4A.

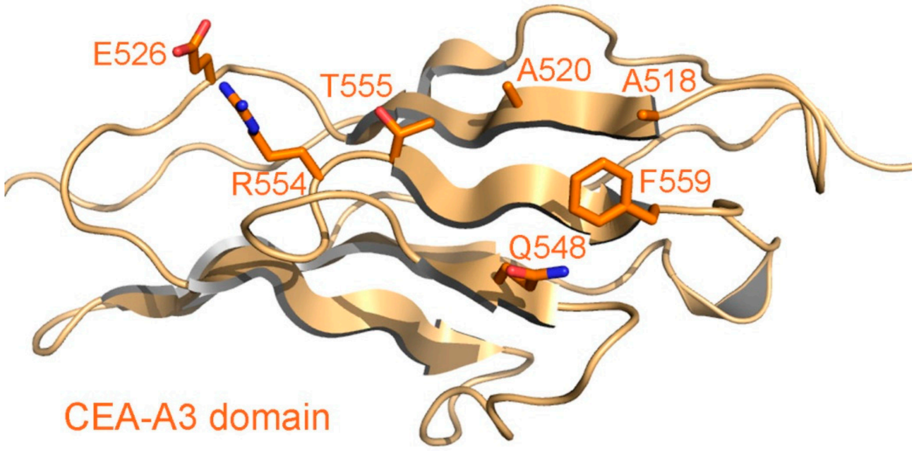

Figure S11: The targeting residues on the A3 domain of the CRC marker CEA for designing the binding protein CEABP1 are shown.

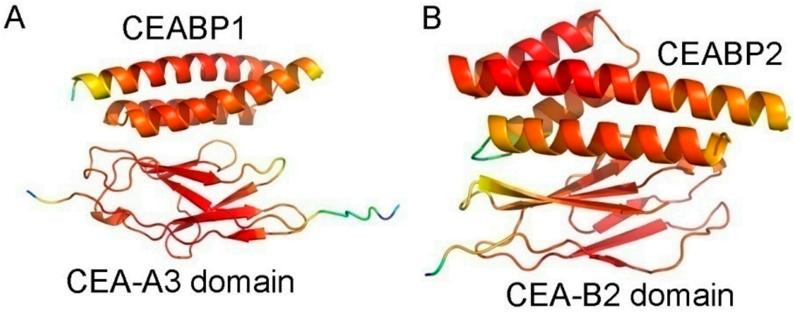

Figure S12: Confidence levels of the predicted structure of CEABP1 in complex with the CEA-A3 domain and that of CEABP2 in complex with the CEA-B2 domain were very high. (A) The predicted local distance difference test (pLDDT) scores of CEABP1 and the A3 domain of CEA were mapped to the predicted structure of their complex. Higher confidence values are shown in warmer colors (red, orange, and yellow). (B) The pLDDT scores of CEABP1 and the B2 domain of CEA were mapped to the predicted structure of their complex. Higher confidence values were shown in warmer colors (red, orange, and yellow).

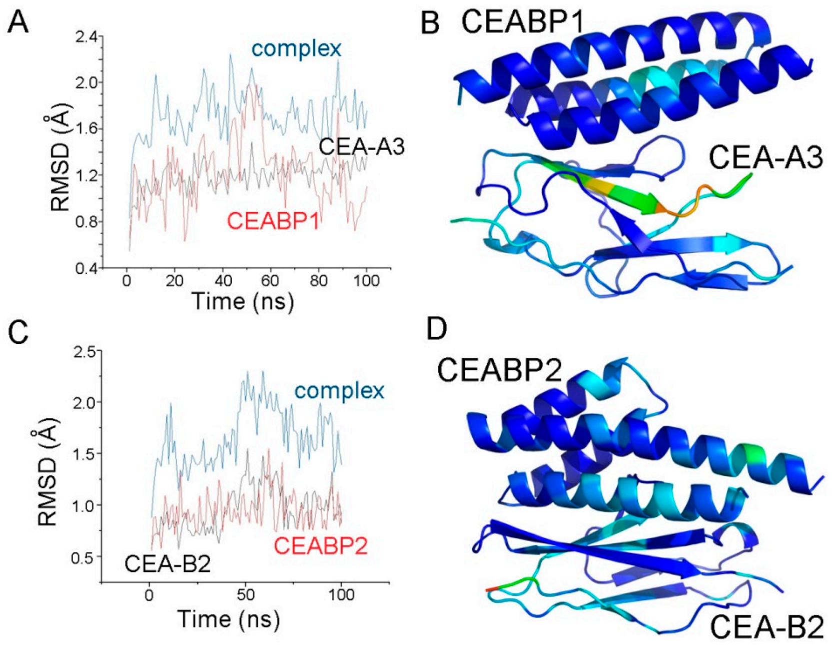

Figure S13: Molecular dynamics simulation showed that the protein complex between CEABP1 and the CEA-A3 domain and that between CEABP2 and the CEA-B2 domain were stable. (A) Time-dependent root mean square deviation (RMSD) of the complex between CEABP1 and the CEA-A3 domain from molecular dynamics simulation. (B) The structure of CEABP1 in complex with the CEA-A3 domain was colored according to the root mean square fluctuation (RMSF) values from the molecular dynamics simulation. Greater fluctuations were shown in warmer colors (red, orange, and yellow) and lower fluctuations were shown in cooler colors (blue and green). (C) RMSD of the complex between CEABP2 and the CEA-B2 domain from molecular dynamics simulation. (D) The structure of CEABP2 in complex with the CEA-B2 domain was colored according to the RMSF values from the molecular dynamics simulation. Greater and lower fluctuations were shown in warmer and cooler colors, respectively.

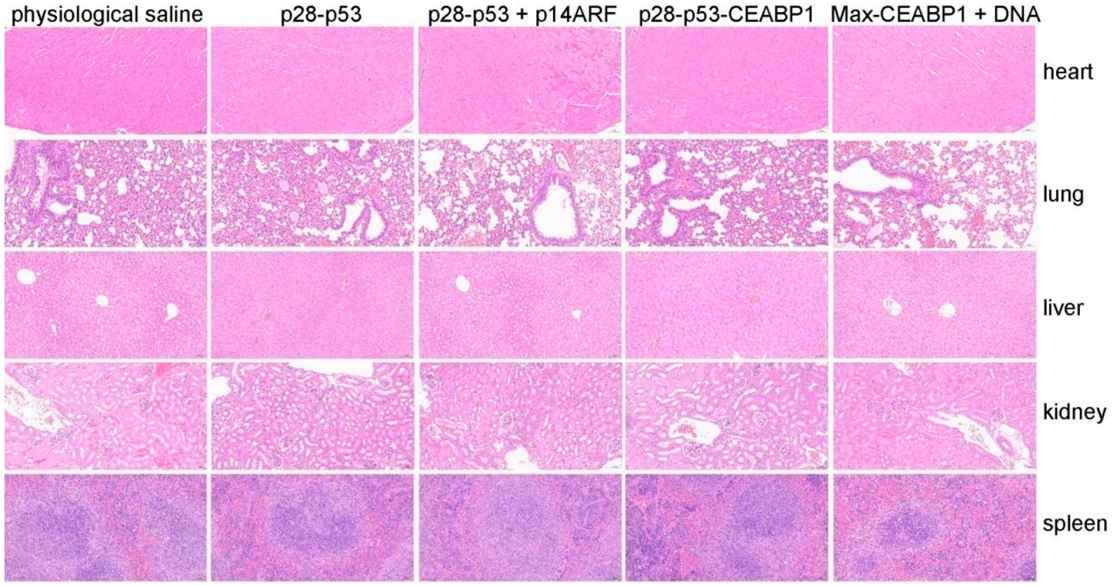

Figure S14: Hematoxylin and eosin (H&E) staining analysis of harvested major organs (heart, liver, spleen, lung, and kidney) revealed no detectable histopathological abnormalities in mice treated with p28-p53 protein, p28-p53 and p28-p14ARF proteins, p28-p53-CEABP1 protein, or pep1-Max-CEABP1 protein together with TCF/LEF TFD DNA.

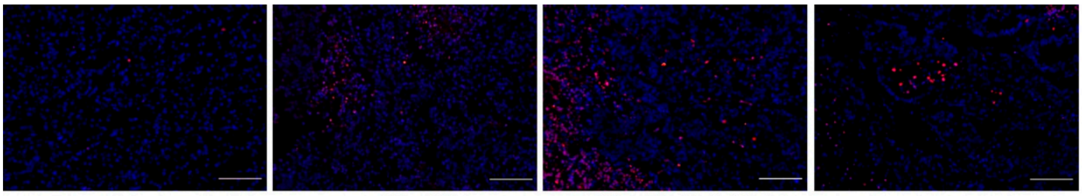

Figure S15: Fusion of p28-p53 with the designed CEABP1 protein targeting the CRC market CEA enhanced the level of apoptosis in the tumor tissues when administered into HCT116 cells xenograft tumor mice. Fluorescence microscopy images of fixed tumor tissues subjected to TUNEL staining. Blue: DAPI; red: apoptotic cells. Scale bars: 100 μm.

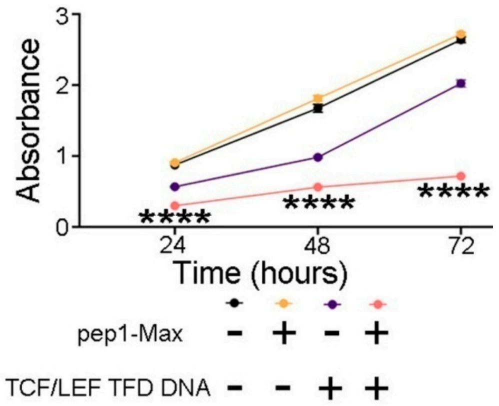

Figure S16: Delivery of TCF/LEF TFD DNA by the purified pep1-Max protein inhibited the proliferation of HCT-116 cells, as revealed by the CCK-8 assay.

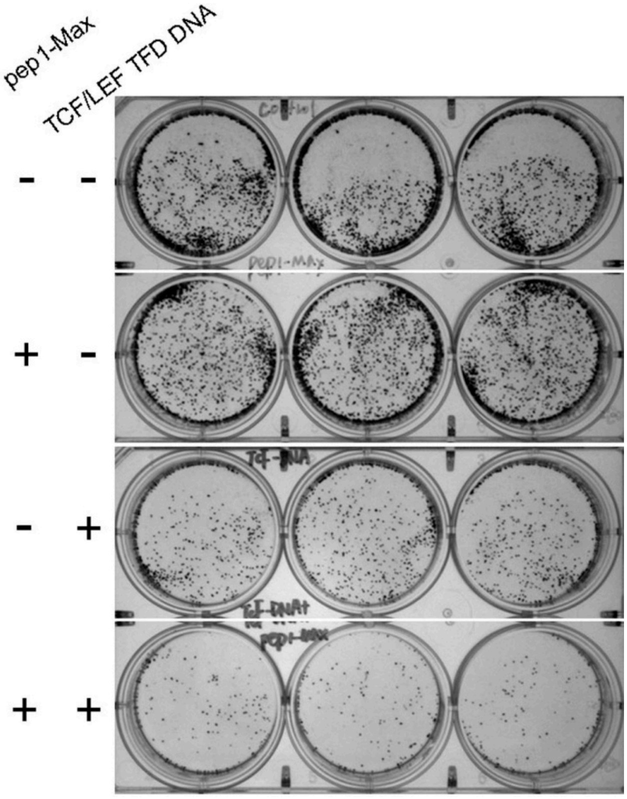

Figure S17: Delivery of TCF/LEF TFD DNA by purified pep1-Max protein suppressed HCT116 cell proliferation in the colony formation assay, corresponding to Figure 9D.

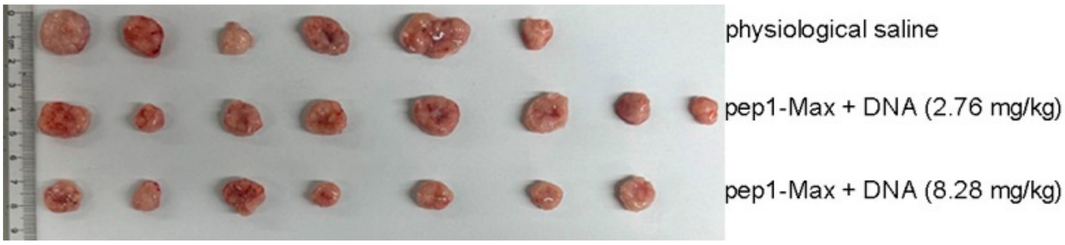

Figure S18: Representative images of HCT116 cells xenograft tumors at the experimental endpoint, which corresponded to Figure 9E.

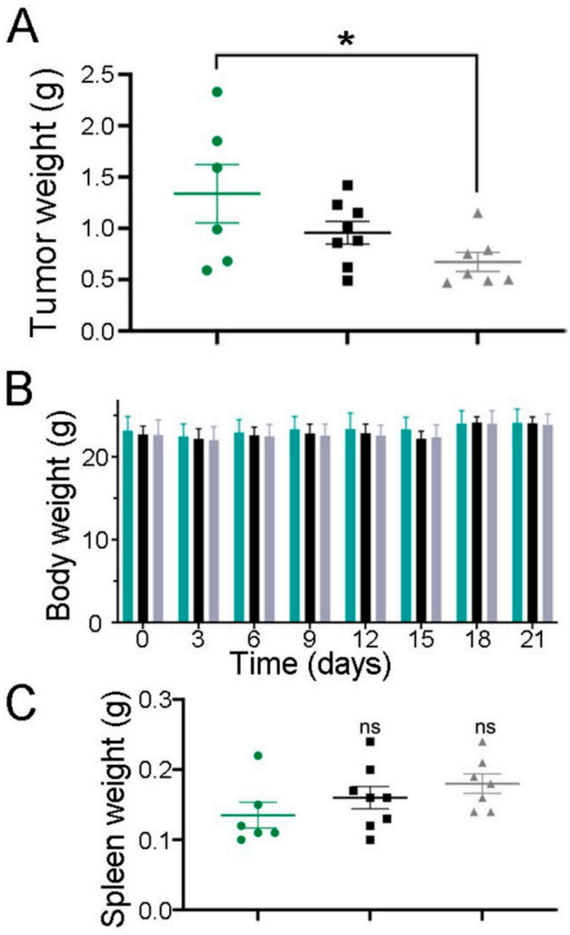

Figure S19: Administration of TCF/LEF TFD DNA and purified pep1-Max protein slowed down the growth of HCT116 cells xenograft tumor but did not affect the body weights and spleen weights of mice. (A) Tumor weights at the experimental endpoint (day 21). The color scheme for different groups was the same as that in Figure 9E. (B) Treatment of mice with TCF/LEF TFD DNA and pep1-Max protein did not affect the body weight of the mice during the experiment. The color scheme for different groups was the same as that in Figure 9E. (C) Treatment of the mice with TCF/LEF TFD DNA and pep1-Max protein did not affect the spleen weight of the mice at the experimental endpoint (day 21). The color scheme for different groups was the same as that in Figure 9E.

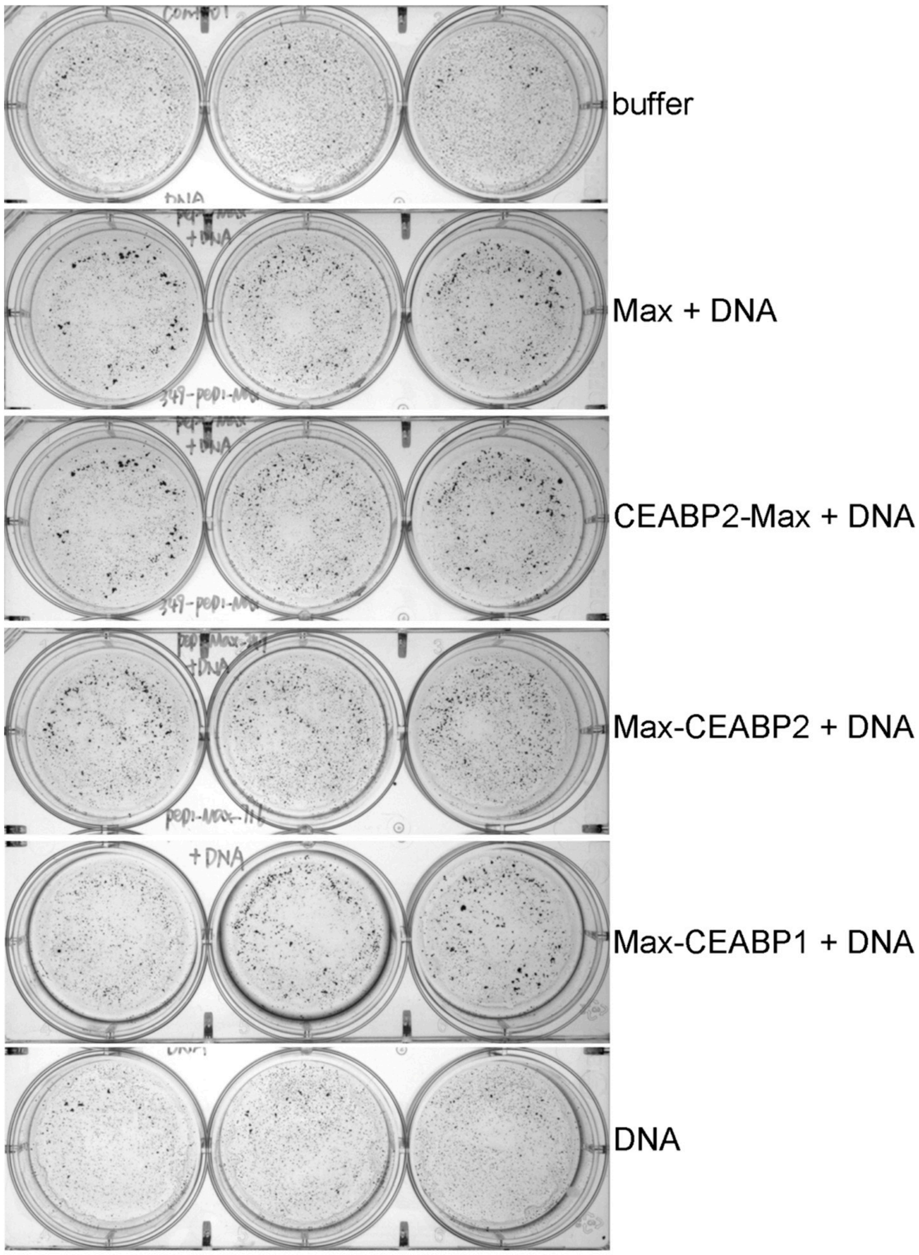

Figure S20: Colony formation assay results showed that fusing CEABP1 to pep1-Max caused the delivered TCF/LEF TFD DNA to inhibit LS174T cell growth more strongly than it did in the absence of CEABP1, which corresponded to Figure 10B.

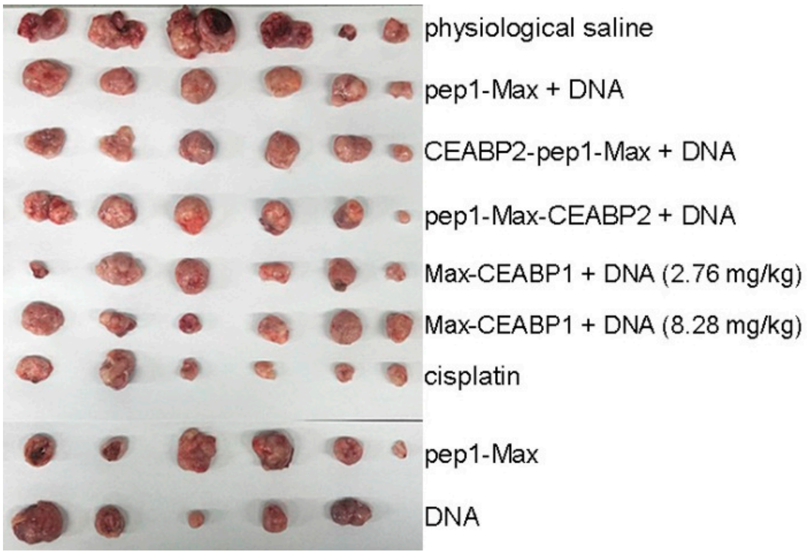

Figure S21: Representative images of LS174T CRC cells xenograft tumors at the experimental endpoint, which corresponded to Figure 10C.

SUPPLEMENTARY MOVIE

Movie S1: The predicted structure model of p14ARF (residues 1-63) in complex with human Mdm2 (residues 186-255) was subjected to molecular dynamics simulation, and the complex was found to be stable during the molecular dynamics simulation process.
